# Mechanical stimulation in wheat triggers age- and dose-dependent alterations in growth, development and grain characteristics

**DOI:** 10.1093/aob/mcab070

**Published:** 2021-06-06

**Authors:** Rebecca Hindhaugh, Maurice Bosch, Iain S Donnison

**Affiliations:** Institute of Biological, Environmental and Rural Sciences (IBERS), Aberystwyth University, Plas Gogerddan, Aberystwyth, UK

**Keywords:** Brushing, grain traits, grain yield, growth and development, mechanical stimulation, mechanical stress, plant morphology, thigmomorphogenesis, touch response, wheat, wind, X-ray micro-computed tomography

## Abstract

**Background and Aims:**

Wheat crops are exposed to a range of mechanical stimulations in their natural environment, yet we know very little about their response to such conditions. The aim of this study was to better understand the effect of mechanical stimulation on wheat growth and development, stem mechanical properties and grain measures. We focused on the following questions: (1) Does plant age affect the response to mechanical stimulation? (2) Is there a minimum threshold for the perception of mechanical stimuli? (3) Is the effect of manual brushing different to natural wind stimulation?

**Methods:**

For age– and dose–response experiments, wheat plants were grown under controlled glasshouse conditions with brushing treatments applied using a purpose-built rig. The results of the controlled experiments are compared with those from an outside experiment where wheat plants were exposed to natural wind, with or without additional brushing. Detailed phenotypic measurements were conducted and treatment effects on grain characteristics were determined using micro-computed tomography imaging.

**Key Results:**

Two-week-old wheat plants were particularly sensitive to mechanical stimulation by controlled brushing treatments. Amongst others, plants exhibited a large reduction in height and grain yield, and an increase in tillers, above-ground biomass and stiffness of stem segments. Plants responded significantly to doses as small as one daily brushstroke. Outdoor experiments by and large confirmed results from controlled environment experiments.

**Conclusions:**

The morphological and developmental response to mechanical brushing treatment, in relation to vegetative above-ground biomass and grain yield, is dependent on plant age as well as the dose of the treatments. This study shows that mechanical stimulation of wheat impacts on a multitude of agriculturally relevant traits and provides a much needed advancement of our understanding of wheat thigmomorphogenesis and the potential applications of mechanical conditioning to control relevant traits.

## INTRODUCTION

The local environmental conditions that plants are exposed to, such as water and nutrient availability, temperature and light, influence their physiology, growth and productivity. Since plants are central to our food production, understanding plant responses to different environmental conditions is a major research area in plant biology.

One environmental condition that has received little attention is mechanical stimulation. Wind is the main cause of mechanical stimulation in the natural environment. Its constant and ambient presence can affect the form and growth of plants, while high wind speeds can cause damage. In addition to wind, rain, movement of animals and other plants, and husbandry practices may also have an effect.

Plant responses to mechanical stimulation are defined by the term thigmomorphogenesis as morphogenic and nastic responses to touch (Jaffe, 1973). While thigmomorphogenic responses can affect a wide range of traits related to plant growth and development, the most commonly observed features are a decrease in shoot elongation and a general reduction in size. These effects appear to be mostly independent of the type of mechanical stimulation (e.g. brushing, bending, shaking or direct exposure to wind). In addition, changes in other morphological traits such as stem diameter, tiller number, leaf size and flowering have been reported (for reviews, see Biddington, 1986; Börnke and Rocksch, 2018). Besides morphological changes, mechanical stimulation can also affect the mechanical properties of the stem (Paul-Victor and Rowe, 2011; Gardiner *et al.*, 2016; Gladala-Kostarz *et al.*, 2020) as well as cell-wall-related features (Roignant *et al.*, 2018; Gladala-Kostarz *et al.*, 2020). As with all environmental stresses, the nature and extent of the responses to mechanical stimuli vary from species to species and are dependent on the developmental stage of the plant during treatment as well as the intensity and duration of treatment (Biddington and Dearman, 1985; Börnke and Rocksch, 2018; Zhao *et al.*, 2018).

The effect of thigmomorphogenesis has been well characterized in dicots and has led to the development of commercial applications of mechanical treatments in the horticultural industry, such as strengthening vegetable seedlings before planting in the field (Mitchell, 1996; Björkman, 1998) or increasing the aesthetic appearance of potted plants (Latimer, 1998). However, the response of monocot grasses (Poaceae or Gramineae) to mechanical stimulation is less well understood (Gladala-Kostarz *et al.*, 2020).

Wheat (*Triticum* sp.) is the most widely cultivated cereal in the world and one of the most important food crops, contributing about 20% of the total dietary calories and proteins worldwide (Shiferaw *et al.*, 2013). Studies on mechanical stimulation in wheat have mostly focused on lodging, which is defined as the permanent displacement of plant stems from their vertical position (Pinthus, 1974) as a result of wind acting on the shoot and rain or irrigation weakening the soil and reducing anchorage strength (Berry *et al.*, 2004). Lodging can lead to significant reductions in grain yield (Berry *et al.*, 2004; Berry and Spink, 2012) and quality (Khobra *et al.*, 2019), and reducing the potential for crops to lodge has therefore been an important target of breeding programmes. The risk of lodging has been reduced by introducing dwarfing genes to produce shorter varieties, by application of plant growth regulators and by optimized crop management practices (Piñera-Chavez *et al*., 2016). The mechanical properties of the stem, dictated amongst others by anatomical features of stem tissues and cell wall composition, also impact on the lodging tolerance (Khobra *et al.*, 2019).

Few studies have examined the effect of controlled mechanical stimulation on wheat plants, or for that matter the grasses in general (Gladala-Kostarz *et al.*, 2020). Rubbing stems for 11 d significantly inhibited the growth of the cereals barley (*Hordeum vulgare*), rye (*Secale cereal*) and maize (*Zea mays*) (by 42, 35 and 28 %, respectively), but wheat plants were not significantly impacted by the rubbing treatment (Jaffe, 1973). No differences in stem height were observed between field-grown wheat plants supported by a frame (preventing wind sway) or free standing (Crook and Ennos, 1996). For centuries, Japanese farmers have been applying mechanical stimulation to young wheat and barley plants, especially at the seedling stage. This process, called ‘mugifumi’, involves the treading of plants and results in plants that are more resilient with higher yields compared to untreated plants (Iida, 2014).

The aim of this study was to better understand the effect of mechanical stimulation on the growth and development of wheat plants, and consequences of mechanical treatments on stem mechanical properties and grain measures. More specifically, we focused on addressing the following three questions: (1) Does plant age affect the response to mechanical stimulation? (2) Is there a minimum threshold for the perception of mechanical stimuli? (3) Is the effect of manual brushing different to natural wind stimulation? Results from age–response experiments, conducted under controlled glasshouse conditions with brushing treatment applied using a purpose-built rig, show that mechanical stimulation particularly induces changes to phenotypic traits, stem mechanical properties and grain measures upon treatment of young wheat plants. Dose–response experiments reveal that plants already respond significantly to doses as small as one daily brushstroke. The results of the controlled experiments are compared with those from an outside experiment where wheat plants were exposed to natural wind, with or without additional brushing. Our findings show that mechanical stimulation of wheat impacts on a multitude of agriculturally relevant traits, providing a much needed and timely advancement of our understanding of the consequences of wheat thigmomorphogenesis and potential applications of mechanical conditioning as an environmentally friendly application to control plant architecture.

## MATERIALS AND METHODS

### Plant material

For experiments in which wind and brushing treatments were compared, the winter wheat variety JB Diego (Senova seeds) was selected. Subsequent experiments (age–response, dose–response and natural conditions) were carried out using the spring wheat variety Mulika (Senova seeds). As this variety does not require vernalization, the effect of treatment on growth, flowering and yield could be studied in a shorter time than for winter wheat. Unless stated otherwise, experiments were carried out under glasshouse conditions: 20 °C day, 10 °C night; natural light supplemented with 10 h light from 400-W sodium lamps. Plants were watered daily, with water added to trays to prevent leaves from becoming wet which could affect the brushing treatment. No fertilizer was used.

### Wind versus brushing assessment

Ninety seeds of JB Diego were sown into 0.5-L pots containing John Innes no. 3 compost, one seed per pot, and watered daily. Two weeks after the seedlings had emerged, 60 plants with similar height were selected and distributed randomly amongst three groups of 20 and assigned a treatment: simulated wind, brushing or left untreated. Wind-treated plants were exposed to simulated wind from a domestic fan (Advent, AVAC 18×; Supplementary Data Movie S1A) for 8 h a day with an average wind speed of 3.5 m s^–1^ measured using an Omega instruments handheld anemometer (Omega, model HHF11A). Daily plant rotations ensured all plants in a group received even treatment from the fan. For brushing treatments, plants were brushed using a purpose-built rig (Movie S1B), with a height-adjustable wooden bar to maintain a constant bending treatment at half the canopy height. Plants were treated once each morning with 20 brushstrokes (one stroke is once forward and once back). The speed at which the brushing treatment was applied was kept constant. Control plants grew in ‘static’ conditions with ambient glasshouse airflow <0.3 m s^–1^. Treatments began 2 weeks after seedling emergence, at which point the majority of plants consisted of two fully emerged tillers. Plants were then treated daily for 4 weeks. Data was collected after 4 weeks of treatment.

### Age–response experiment

Three batches of 120 wheat seeds of the variety Mulika were sown into 1.5-L pots containing John Innes no. 3 compost, each batch was sown 2 weeks apart, ensuring that plants would be 2, 4 and 6 weeks post-emergence at the beginning of treatment. When plants reached the treatment stage, 60 similar sized plants from each of the three batches were selected and randomly distributed into six groups of ten plants for each age. Each age group had three groups of treated plants and three groups of untreated plants. Treatment consisted of brushing aerial plant parts with 20 brushstrokes using the purpose-built rig as described before, once per day for 4 weeks. Untreated control plants were grown alongside the treated plants. After 4 weeks of treatment, phenotypic data were collected (T1) and plants were left to continue growing and further measurements were taken when the plants had finished flowering (decimal growth stage 69; Tottman, 1987; AHDB, 2018; T2). Final measurements were taken when plants were fully senesced (T3).

### Dose–response experiment

In total, 350 Mulika seeds were planted as described previously for the age–response experiment and 192 uniformly sized plants were selected 2 weeks after emergence and randomly assigned to groups. Each group consisted of eight plants, with three groups per treatment, therefore totalling 24 plants per treatment. Treatment involved brushing the plants with the purpose-built rig, but now the number of daily brushstrokes applied was one, three, six, nine, 12, 15 or 20. An additional 24 plants, split into three groups, received no treatment. Treatments began 2 weeks after seedling emergence and lasted 4 weeks. Phenotypic data were collected after four weeks of treatment (T1) when the plants had reached decimal growth stage 31 (DGS 31; Tottman, 1987; AHDB, 2018), when they finished flowering (T2; DGS 69), and when they became fully senesced (T3).

### Outdoor experiment

In total, 160 Mulika seeds were planted as described previously for the age–response experiment. Two weeks after emergence, 72 homogenous plants that were uniform in size were selected and randomly distributed across nine groups (three groups per treatments) of eight plants each, and each group was assigned to one of three treatments: static, wind and wind + brushed. Plants were moved to an outside area of hard standing. Each group of eight plants assigned to the static treatment was surrounded with a purpose-built baffle to reduce airflow around and between the plants. Each individual plant within the group was also staked to a basket-like frame in order to further reduce plant movement and leaf flutter. Both the wind-treated and wind + brushed-treated plants remained unsupported and thus were able to sway and move under natural wind conditions. Wind + brushed plants received an additional brushing treatment of 20 brushstrokes once per day using the same rig as described before. Treatments began 2 weeks after seed germination and lasted for 4 weeks. Additional brushing was halted, and plants were left to grow under natural conditions until they had finished flowering (DGS 69). Phenotypic measurements were taken at the three different developmental stages as described before.

### Phenotypic measurements

Stages of growth were determined according to the Zadok’s scale and the AHDB Wheat growth guide (Zadoks *et al.*, 1974; Tottman, 1987; AHDB, 2018). Once plants reached the three-tiller stage, the main tiller was tagged. Plant height at the end of the treatments was measured as the distance from the soil surface to the highest point of the plant. Similar measures were taken for the height of the main tiller at the end of flowering. Tiller numbers were counted at the end of treatments. Internodes were numbered from bottom to top and the length and diameter of each internode were measured. Internode diameters were measured at six locations along each internode using callipers and averaged. Leaf measurements included leaf length, width and area. Leaf length was measured along the centre of each leaf, from the ligule to the tip. Width was measured at the widest section of the leaf. For an estimation of leaf area, the following equation was used, *a* = *l* × *w* × *k*, where *l* is length, *w* is width and *k* is a constant, 0.75 (Bell and Fischer, 1994; Chanda and Singh, 2002). For above-ground biomass dry weight measurements, plants were cut at their base and dried in an oven at 60 °C for 48 h before weighing. The procedure for determining below-ground biomass dry weight is described in Supplementary Data Methods S1.

### Spike-related measures

Fully emerged spikes on each plant were counted. Spike length was measured at the senesced stage as the length from the base of the rachis to the top of the terminal spikelet, excluding any awns. Spike weight was also determined at maturity. The spikes on each plant were labelled and cut from the stem. The main tiller spike from each plant was then weighed individually, and together with all other spikes from the same plant to determine total spike weight.

### CT scanning and grain analysis

Main tiller spikes harvested from senesced plants were analysed using X-ray micro-computed tomography (µCT). Spikes were cut into two equal sections in order to fit into the CT scanner. Samples were loaded into individual holders and scanned using a μCT1000 scanner (Scanco medical, Switzerland); for detailed information on the conditions used and features extracted, see Hughes *et al.* (2017). MATLAB-based software developed by Hughes *et al*. was used to perform feature extraction and is available from github at https://github.com/NPPC-UK/microCT_grain_analyser. Features extracted included individual grain length, width, depth, volume and surface area. Additional information yielded by the software included three-dimensional position of each grain in the spike using *x*, *y*, *z* axis position. Data for each half of separated spikes were recombined to give an output for an entire spike.

### Stem mechanical properties

Main tillers collected at the senesced stage were used for mechanical testing. All leaves and the leaf sheath were stripped from the tiller to leave just the stem. Internode sections were subjected to a three-point bending test using a mechanical texture analyser (TA.XT plus, Stable Micro Systems) equipped with a 50-N loading cell. Exponent-TEE32 software produced a graph detailing force applied to the material (N), and deflection (mm) of the material. After the tests, senesced samples were stored until cross-section measurements could be acquired. Images of stem cross-sections from close to the site of impact were taken using a Leica MZ6 microscope and attached Nikon Coolpix 990 camera. Images were then analysed using ImageJ (Schneider *et al.*, 2012) to calculate external diameter, external radius, internal diameter, internal radius and stem thickness (internal boundary to external boundary transect) where samples were hollow. Pith-filled samples were measured for external diameter and radius. Data from the three-point bending and cross-section measurements were then used to calculate the Young’s modulus using the equations based on Crook and Ennos (1996) and Gordon (2009) as described in Supplementary Data Methods S2.

### Statistical analysis

All analysis was performed using SPSS statistics (2017, IBM SPSS Statistics for Windows, Version 25.0; IBM Corp., Armonk, NY, USA). Treatment effect was analysed using analysis of variance (ANOVA) at the 5 % level (*P* < 0.05) of significance. Where ANOVA indicated a statistically significant difference, post-hoc tests using Tukey’s HSD and Dunnett’s *t* were conducted. Tukey tests conducted pairwise tests between all treatments, including control, while Dunnett’s *t* tests compared each treatment against the control/untreated plant data. Where treatment effect on plants of different ages was compared, *t*-tests were used to compare treated and untreated plants of each age group at the end of treatment.

## RESULTS

### Wind and brushing induce similar phenotypic responses in wheat

To evaluate if mechanical stimulation by brushing induced similar phenotypic responses to wind treatment, wheat plants (variety JB Diego) were either treated daily with 20 brushstrokes using a purpose-built rig or exposed to wind in the form of fan-induced air movement (see Material and methods and Supplementary Data Movie S1). A summary of the results obtained after 4 weeks of treatment is shown in Table S1. Plant height was reduced by both treatments when compared with untreated static control plants, although this reduction was only significant for the wind treatment. The number of tillers, leaves and biomass measures showed a significant increase for both treatments while the length of the main tiller top leaf was significantly reduced (Table S1). These findings showed that mechanical stimulation of 2-week-old wheat seedlings by brushing and wind similarly affected the phenotypic parameters evaluated. Since brushing utilizing the rig allowed for a more controllable, even and reproducible treatment for larger numbers of wheat plants, brushing was used in subsequent mechanical stimulation experiments.

### Age–response

We next wanted to investigate the extent to which the phenotypic response to mechanical stimulation of wheat is affected by its developmental stage. As we also wanted to establish the effect of mechanical stimulation on reproductive measures, an age–response experiment was conducted using the Mulika variety, which does not require vernalization. Plants were brushed (20 strokes) for 4 weeks starting either 2, 4 or 6 weeks post-emergence.

**Fig. 1.**
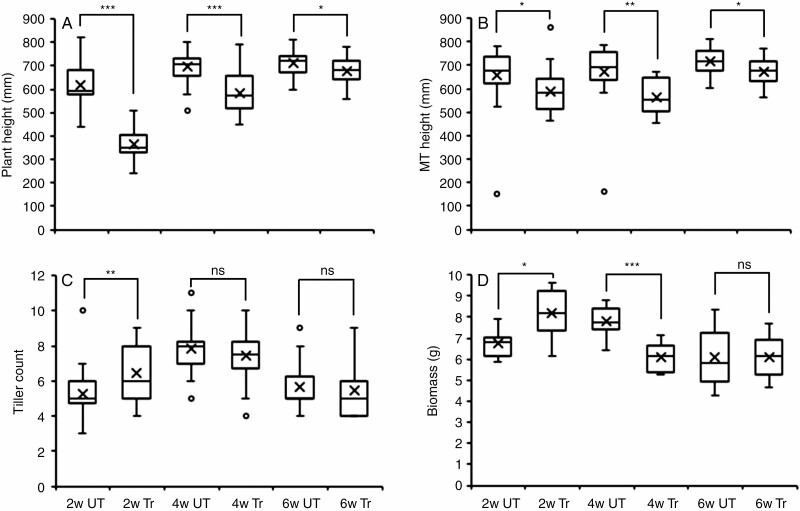
Age-dependent phenotypic effects of brushing treatments on wheat plants. Wheat plants (Mulika variety) of different ages (2, 4 and 6 weeks post-emergence) were brushed daily (1 × 20 brush strokes) for 4 weeks. (A) Plant height measured at the end of treatment (T1). (B) Main tiller height at the end of flowering (T2). (C) Tiller number at the end of treatment (T1). (D) Above-ground biomass of senesced mature plants after removal of the spikes. Significant differences to untreated controls (*t* test) are indicated by asterisks (*P < 0.05, **P < 0.01, ***P < 0.001; ns, not significant). For A *n* = 30, B *n* = 20, C *n* = 30, and D *n* = 10. UT, untreated control; Tr, treated with 20 brushstrokes; MT, main tiller.

*Mechanical stimulation particularly affects the growth and development of young wheat plants.* Although brushing treatments significantly reduced plant height for all age-groups when compared to their respective controls when measured at the end of the treatments, there was a clear age–response effect (Fig. 1A). The largest height reduction (41 %) was observed for the 2-week-old age-group, followed by the 4-week and 6-week age-groups (height reductions of 16 and 5 %, respectively). The length of the main tiller, measured at the end of flowering, was also significantly reduced across the age-groups, although with no clear age–response effect (reductions of 11, 16 and 6 %, respectively for the 2-, 4- and 6-week age-groups) (Fig. 1B). The number of tillers was only significantly affected for the 2-week-old plants, with brushing on average resulting in the production of one more tiller when compared with untreated control plants (Fig. 1C). Above-ground biomass dry weight measures also showed a significant increase for the 2-week age-group only (21 %), while there was a significant decrease of 22 % upon treatment of the 4-week group when compared with their controls with no significant differences in above-ground biomass between the 6-week treated and control plants (Fig. 1D). The flag leaf area of the main tiller was only significantly decreased after treatment of the 2-week age-group, with no effect on leaf area for the other two age-groups (Supplementary Data Table S2). These results suggest that the positive contribution of the increased tiller number to the observed increases in above-ground biomass, triggered by mechanical stimulation of 2-week-old plants, offsets the negative contribution of reduced stem elongation and leaf area in this age-group.

To evaluate a possible association between mechanical stimulation, internodal length and developmental stage, internodal lengths were measured at the end of flowering (Supplementary Data Table S3). Early stimulation at 2 weeks significantly reduced the length of internodes 1, 2 and 3, with a reduction of 53 % (~3 cm), 33 % (~3 cm) and 13 % (~2 cm), respectively, when compared with their respective untreated controls. When treatment commenced when plants were 4 weeks old, internodes 2, 3 and 4 were significantly reduced in their length by 17, 29 and 17 %, respectively, when compared with their respective controls (Table S3). For 6-week-old plants, a significant reduction in length was only observed for internodes 3 and 4 (12 and 8 % reduction, respectively). Those internodes that showed a significant reduction in length also exhibited a significant reduction in their diameter (Table S3), except for 6-week-old plants where none of the internodal diameters were significantly different from their controls.

To assess if brushing affected the mechanical properties of the stems, we determined the Young’s modulus of the third internode of senesced mature plants with a three-point bending test. Mechanical stimulation only affected the mechanical properties of 2-week-old plants, resulting in a significantly increased Young’s modulus (almost double) of brushed plants when compared with untreated controls (Table S4).

**Fig. 2.**
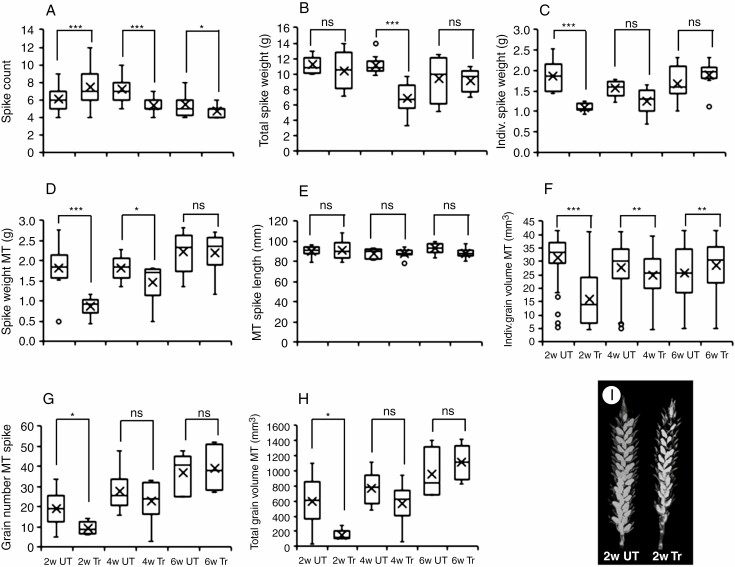
Age–response of spike and grain characteristics after brushing. (A) Average number of flowering spikes per plant at the end of flowering (T2, *n* = 20). (B) Mean total plant spike weight at maturity when plants had become senesced (T3, *n* = 10). (C) Mean individual spike weight at maturity when plants had become senesced (*n* = 10). (D) Weight of mature senesced main tiller spikes (*n* = 10). (E) Length of mature main tiller spikes (*n* = 10). (F) Average individual grain volume of grains in main tiller spikes (*n* = 6). (G) Mean number of grains in main tiller spikes (*n* = 6). (H) Total volume of grains in each main tiller spike (*n* = 6). (I) Stitched micro-CT images of main tiller spike from a 2-week age-group; control (left) and brushing treatment (right). Significant differences to untreated controls (*t* test) are indicated by asterisks (*P < 0.05, **P < 0.01, ***P < 0.001; ns, not significant). 2w, 4w, 6w refer to the age-groups of plants when treatments started. UT, untreated control; Tr, treated with 20 brushstrokes; MT, main tiller.

*Mechanical stimulation affects spike and grain characteristics.* Mechanical stimulation by brushing significantly affected the total number of spikes per plant. Treatments of 2-week-old plants significantly increased the total number of spikes per wheat plant while there was a significant decrease for this measure when plants were older before treatments commenced (Fig. 2A). Although treatments reduced the total weight of all spikes per plant for all the age-groups, this decrease was only significant for the 4-week-old age-group where brushing reduced this measure by almost 40 % (Fig. 2B). The average weight of individual spikes was only significantly reduced for 2-week-old plants (42 %, *P* < 0.001) when compared with their untreated controls. No significant effects for the other two age-groups were observed (Fig. 2C). These results indicate that brushing 2-week-old wheat plants increases the number of spikes, probably as a result of an increase in tiller number (Fig. 1C), but not the total spike weight, thus resulting in a lower weight of individual spikes. Brushing 4-week-old plants reduced both the number of spikes and the total spike weight, not significantly affecting the weight of individual spikes. Brushing 6-week-old plants had little effect on spike measures.

To examine the effect of brushing on spike development further, the spikes of the main tillers were analysed in more detail. In accordance with the average spike weight (Fig. 2C), the weight of mature main tiller spikes was also significantly reduced for the 2-week age-group (a decrease in weight by ~50 % when compared with controls) with a ~15 % decrease (*P* = 0.045) for the 4-week group and no treatment effect for the 6-week group (Fig. 2D). Brushing did not have a significant effect on main tiller spike length for any of the ages (Fig. 2E).

µCT is a non-invasive and non-destructive method which can yield detailed three-dimensional images of internal structures based on differential X-ray penetration of materials of differing compositions and densities. Here we used µCT imaging of the main tiller spikes to assess the effect of mechanical stimulation on grain characteristics *in situ* in the spike (Fig. 2I). The average volume of individual grains followed a similar trend as the spike weight on the main tiller, with a ~50 % decrease (*P* < 0.001) in grain volume for the treated 2-week group when compared with controls (Fig. 2F). A modest, but significant, decrease (10 %) and increase (11 %) was observed for the 4- and 6-week age-groups, respectively. Further morphometric feature extraction showed that the reduction in grain volume for the treated 2-week group was mainly caused by a reduction in grain width and depth (Table 1). The number of grains on the main tiller followed a similar pattern as seen for the volume, with a significant decrease (~50 %) in grain number for the 2-week group (Fig. 2G), and decrease and increase for the 4- and 6-week age-groups, respectively (both not significant). Combining the results for average grain volume with grain number resulted in an almost 4-fold reduction of total grain volume in the main tiller spikes for the 2-week group (Fig. 2H). The observed decrease in total grain volume for the 4-week group and increase for the 6-week group were not significant.

**Table 1.** Morphometric traits of main tiller grains from the age–response experiment

		*n*	Grain length (mm)	Grain width (mm)	Grain depth (mm)
2 Weeks	Untreated	115	4.94 ± 0.086	3.92 ± 0.059	3.05 ± 0.044
	Treated	57	4.72 ± 0.198	3.01 ± 0.112	2.10 ± 0.074
			*P* = 0.301	*P* < 0.001	*P* < 0.001
				***	***
4 Weeks	Untreated	166	4.79 ± 0.064	3.81 ± 0.065	2.91 ± 0.041
	Treated	137	5.16 ± 0.077	3.41 ± 0.063	2.62 ± 0.041
			*P* < 0.001	*P* < 0.001	*P* < 0.001
			***	***	***
6 Weeks	Untreated	223	4.87 ± 0.048	3.51 ± 0.059	2.72 ± 0.044
	Treated	235	4.94 ± 0.042	3.67 ± 0.053	2.88 ± 0.035
			*P* = 0.235	*P* = 0.050	*P* = 0.004
					**

Significant differences to untreated controls are indicated by asterisks (**P < 0.01, ***P < 0.001).

Data in this table are means ± standard error.

Together, these results indicate that brushing has a significant impact when imposed on 2-week-old wheat plants, resulting in lower grain yield.

### Dose–response

Having established that mechanical stimulation of 2-week-old wheat plants had the strongest effect on phenotypic development and grain measures, we next assessed if there was a dose–response to brushing treatments for plants of this age-group. To examine this, 2-week-old wheat plants were brushed daily for 4 weeks with a range of doses: one, three, six, nine, 12, 15 and 20 brushstrokes per day.

**Fig. 3.**
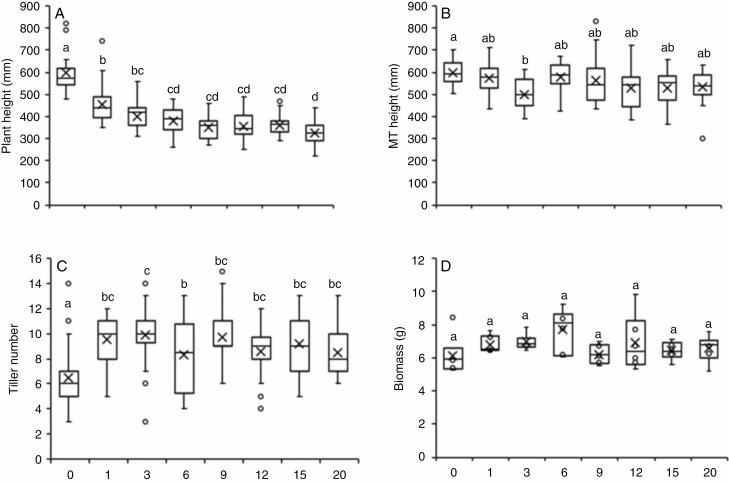
The effect of different doses of brushing treatments on phenotypic measures in wheat plants. Two-week-old wheat plants were brushed with doses ranging from 0 to 20 daily strokes for 4 weeks. (A) Plant height measured at the end of treatment (T1). (B) Main tiller height at the end of flowering (T2). (C) Tiller number at the end of treatment (T1). (D) Above-ground biomass of senesced mature plants after removal of the spikes. ANOVA with a post-hoc Tukey test was performed to identify statistical differences (*P* ≤ 0.05) indicated by lower case letters. The number of daily brushstrokes is indicated on the *x*-axis. For A, B and C, *n* = 24; for D, *n* = 6. MT, main tiller.

*A few brushstrokes trigger the main phenotypic changes.* The application of just one brushstroke per day for 4 weeks reduced the plant height by 146 mm (24 %) compared with untreated plants at the end of the treatment. Increasing the number of daily brushstrokes reduced plant height further (Fig. 3A) with the mean plant height after 20 strokes again being significantly lower (by 28 %) compared with just one daily brushstroke and 46 % lower compared to untreated controls.

The main tillers showed a similar trend when their height was measured at the end of flowering, with brushing resulting in a reduction of main tiller height, although these reductions were not always significant (Fig. 3B). When looking at the length of the individual internodes, the number of brush strokes significantly affected the bottom two internodes, with even one daily brushstroke significantly reducing the length of internode 1 by 44 % compared with untreated plants, while this height reduction was 58 % for 20 brushstrokes (Supplementary Data Table S5). For internode 2, the reduction in height was 27 and 40 % for one and 20 brushstrokes, respectively. A Tukey HSD post-hoc test indicated that there was no significant difference in the length of internode 2 between plants that received more than three brushstrokes. Overall, treatments did not significantly affect the length of the third and fourth internodes. Although treatments resulted in the reduction of the diameter for some internodes (Table S5), no particular dose–response could be observed.

As seen before for the age–response for the 2-week age-group, 20 brushstrokes significantly increased the number of tillers per plant (Fig. 3C). Remarkably, even one daily brushstroke resulted in a significantly increased tiller number not distinct from 20 brushstrokes, or any of the other doses.

**Fig. 4.**
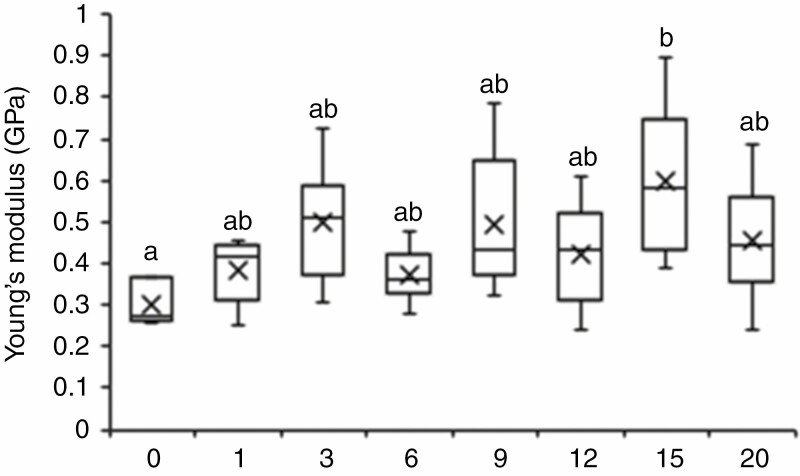
Increases in the Young’s modulus of stem segments from senesced mature plants are not dose-dependent. Numbers on the *x*-axis indicate the number of daily brushstrokes. ANOVA with a post-hoc Tukey test was performed to identify statistical differences (*P* ≤ 0.05) indicated by lower case letters.

Above-ground biomass was increased for all the dose–response treatments compared with controls (Fig. 3D), although there was no correlation between dose and biomass increase and none of the increases was statistically significant. The highest increase was observed for six brushstrokes (25 %) and the lowest for nine brushstrokes (1 %) with one and 20 brushstrokes increasing the biomass by 11 and 7 %, respectively, when compared with controls. All of the doses affected the mechanical properties of senesced stems with an increase in the Young’s modulus of internode 3 ranging from 23 to 99 % compared with untreated control plants (Fig. 4).

Together, these phenotypic measurements show that a few daily brushstrokes (one or three) generally trigger the same phenotypic effects as for 20 brushstrokes, suggesting that saturation of the response to mechanical stimulation is achieved with just a few brushstrokes.

*A few brushstrokes affect spike and grain characteristics.* Looking at the dose–response effect on reproductive measures, a significant increase in the number of spikes compared with untreated controls was observed for most dose treatments, with no obvious dose–response effect. The highest increase in spike count was observed for three daily brushstrokes (42 % increase) and the lowest for 12 strokes (16 % increase), with a 21 and 25 % increase for one and 20 daily brushstrokes, respectively (Table 2). The latter was very similar to what was observed when 20 brushstrokes were applied to 2-week-old plants for the age–response experiment (Fig. 2A).

**Table 2.** Spike and grain measurements from dose–response experiments

Treatment (no. of brushstrokes)		0	1	3	6	9	12	15	20
**Number of flowering spikes per plant**									
	*n* = 24	6.04 ± 0.332	7.29 ± 0.29	8.56 ± 0.34	7.53 ± 0.32	7.5 ± 0.27	7.00 ± 0.32	7.58 ± 0.25	7.54 ± 0.34
			*P* = 0.018	*P* < 0.001	*P* = 0.012	*P* = 0.012	*P* = 0.116	*P* = 0.002	*P* = 0.003
			*	***	*	*		**	**
		a	abc	c	bc	bc	ab	bc	bc
**Average spike weight per plant (g)**									
	*n* = 6	1.79 ± 0.07	1.31 ± 0.07	1.29 ± 0.05	1.39 ± 0.04	1.23 ± 0.08	1.22 ± 0.11	1.22 ± 0.10	1.23 ± 0.04
			*P* < 0.001	*P* < 0.001	*P* = 0.003	*P* < 0.001	*P* < 0.001	*P* < 0.001	*P* < 0.001
			***	***	**	***	***	***	***
		a	b	b	b	b	b	b	b
**Total spike weight (g)**									
		11.25 ± 0.85	10.08 ± 0.23	11.28 ± 0.31	11.05 ± 0.64	9.87 ± 0.56	9.22 ± 0.96	10.44 ± 0.57	9.79 ± 0.85
			*P* = 0.693	*P* = 1.000	*P* = 1.000	*P* = 0.540	*P* = 0.172	*P* = 0.920	*P* = 0.476
		a	a	a	a	a	a	a	a
**T3 Main tiller spike weight (g)**									
		1.88 ± 0.10	1.41 ± 0.08	1.12 ± 0.16	1.45 ± 0.05	1.25 ± 0.10	0.97 ± 0.16	1.07 ± 0.14	0.98 ± 0.10
			*P* = 0.034	*P* < 0.001	*P* = 0.066	*P* = 0.003	*P* < 0.001	*P* < 0.001	*P* < 0.001
			*	***		**	***	***	***
		a	ab	b	ab	b	b	b	b
**Total grain volume (mm**^**3**^)									
		898 ± 105.1	339 ± 77.3	318 ± 90.6	250 ± 51.1	305 ± 85.3	215 ± 35.4	147 ± 38.5	218 ± 61.6
			*P* < 0.001	*P* < 0.001	*P* < 0.001	*P* < 0.001	*P* < 0.001	*P* < 0.001	*P* < 0.001
			***	***	***	***	***	***	***
		a	b	b	b	b	b	b	b
**Grain count**									
		33.6 ± 3.99	14.2 ± 2.91	12.7 ± 3.20	10.2 ± 1.85	12.8 ± 3.53	9.2 ± 1.40	7.5 ± 1.18	10.7 ± 2.29
			*P* < 0.001	*P* < 0.001	*P* < 0.001	*P* < 0.001	*P* < 0.001	*P* < 0.001	*P* < 0.001
			***	***	***	***	***	***	***
		a	b	b	b	b	b	b	b
**Average grain volume (mm^3^)**									
		26.8 ± 0.39	23.9 ± 1.10	25.0 ± 0.93	24.6 ± 1.28	23.7 ± 0.98	23.5 ± 1.59	19.6 ± 1.52	20.4 ± 1.15
			*P* = 0.122	*P* = 0.750	*P* = 0.609	*P* = 0.090	*P* = 0.146	*P* < 0.001	*P* < 0.001
								***	***
		a	abc	a	ab	abc	abc	c	bc
**Grain length (mm)**									
		4.97 ± 0.038	5.01 ± 0.113	5.08 ± 0.120	4.90 ± 0.142	5.05 ± 0.108	4.59 ± 0.161	5.00 ± 0.187	4.98 ± 0.139
			*P* = 1.000	*P* = 0.935	*P* = 0.998	*P* = 0.991	*P* = 0.038	*P* = 1.000	*P* = 1.000
							*		
		ab	ab	b	ab	ab	a	ab	ab
**Grain width (mm)**									
		3.62 ± 0.041	3.36 ± 0.077	3.45 ± 0.079	3.50 ± 0.106	3.47 ± 0.104	3.49 ± 0.129	3.01 ± 0.122	3.12 ± 0.094
			*P* = 0.056	*P* = 0.468	*P* = 0.894	*P* = 0.629	*P* = 0.856	*P* < 0.001	*P* < 0.001
								***	***
		a	abc	ab	ab	ab	ab	c	bc
**Grain depth (mm)**									
		2.81 ± 0.025	2.66 ± 0.058	2.67 ± 0.055	2.67 ± 0.064	2.53 ± 0.059	2.68 ± 0.077	2.32 ± 0.089	2.30 ± 0.063
			*P* = 0.082	*P* = 0.172	*P* = 0.299	*P* < 0.001	*P* = 0.398	*P* < 0.001	*P* < 0.001
						***		***	***
		c	bc	bc	bc	ab	bc	a	a

Significant differences to untreated controls are indicated by asterisks (*P < 0.05, **P < 0.01, ***P < 0.001).

ANOVA with a post-hoc Tukey test was performed to identify statistical differences (*P* < 0.05), indicated by lower case letters.

Data in this table are means ± standard error.

Although the total spike weight per plant for the different doses was mostly lower compared with untreated controls, these decreases were not significant (Table 2). However, the average weight of individual spikes was significantly reduced for all the doses when compared with untreated controls (Table 2) with no clear dose–response effect. The decrease in spike weight was 27 % for one brushstroke and 31 % for 20 brushstrokes.

In agreement with the reduced weight of the individual spikes per plant for all the doses, the weight of main tiller spikes was significantly reduced compared with controls (Table 2). µCT analysis of the main tiller spikes revealed a general decline in the volume of individual grains, although this was only significant for 15 and 20 brushstrokes (Table 2). As also observed for the age–response, the volume reductions were mainly caused by decreases in grain width and depth (Table 2). All dose treatments resulted in a significant reduction of the number of grains per spike at maturity, ranging from a 58 % reduction with one stroke to a 78 % reduction with 15 strokes and 68 % for 20 strokes, with no significant differences between the dose treatments (Table 2). Since brushing led to both a decrease in the volume of individual grains and a decrease in the number of grains, the total grain volume for each main tiller spike at maturity was significantly reduced, by more than half on average, ranging from a 60 % reduction for one brushstroke to a 6-fold reduction for 15 brushstrokes (Table 2). Again, there was no significant difference between the total spike grain volumes between the different dose treatments (ranging from one to 20 brushstrokes).

In summary, these results show that even one daily brushstroke significantly affects spike and grain measures when applied to 2-week-old wheat plants.

### Effect of wind exposure and brushing on wheat plants grown under natural conditions

The results described thus far were all obtained under controlled environmental conditions. To start evaluating the effect of mechanical stimulation when plants are exposed to natural conditions, a pot experiment was devised where wheat plants were placed outside 2 weeks post-emergence and exposed to two different treatments: unsupported and exposed to natural wind; and unsupported exposed to natural wind with additional mechanical treatment (20 brushstrokes per day for 4 weeks). Control plants were supported and protected with baffles (Supplementary Data Fig. S1) to reduce airflow around and between plants.

**Fig. 5.**
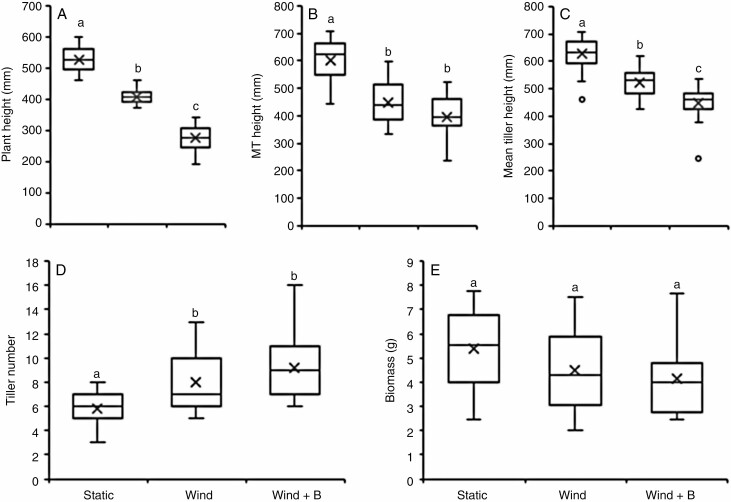
The effect of natural conditions on wheat phenotypic measures. Two-week-old wheat plants were either exposed to natural wind (Wind), or the former plus additional 20 daily brushstrokes for 4 weeks (Wind + B). Control plants were shielded from wind (Static). (A) Plant height measured at the end of treatment (T1). (B) Main tiller height at the end of flowering (T2). (C) Mean height of tillers at the end of flowering (T2). (D) Tiller number at the end of treatment (T1). (E) Above-ground biomass of senesced mature plants after removal of the spikes. ANOVA with a post-hoc Tukey test was performed to identify statistical differences (*P* ≤ 0.05) indicated by lower case letters. For A–D, *n* = 24; for E, *n* = 12. MT, main tiller.

*Mechanical stimulation under pseudo-natural conditions affects above-ground biomass.* The two treatments induced a significant reduction in plant height at the end of treatment (Fig. 5A) and main tiller height at the end of flowering (Fig. 5B). Exposure to wind reduced plant height and main tiller length by 23 and 25 % at these two stages, respectively, while additional brushing further reduced these height measures by 48 and 34 %, respectively, when compared with the height of static control plants at these two developmental stages. The mean height across all tillers at the end of flowering also showed a significant reduction for both treatments when compared with controls (Fig. 5C). Wind exposure reduced average tiller height by 17 % with additional brushing causing a further decrease, reducing height by 29 % compared with controls. Additional measurements, including for base to flag leaf and distance between auricle to ligule (Supplementary Data Table S6) corroborated findings for tiller height data.

Length measurements of individual internodes showed that internodes 2, 3 and 4 were all significantly reduced in length for both treatments compared with controls (Supplementary Data Table S7). The base internode, internode 1, which was 30 mm long in the control group (although only present in 11 out of the 24 plants), could not be identified after both treatments, suggesting that this internode was severely reduced in size or did not develop at all.

The diameters of the internodes of treated plants were mostly not significantly different from the control group, except for internodes 2 and 3 after wind treatments, which showed a significant decrease in diameter although there was no significant difference in the internode diameters between wind and wind + brushing treatment (Supplementary Data Table S7).

Both treatments significantly increased the number of tillers, from an average of 5.8 tillers in control plants to 8.0 tillers after wind treatment and 9.2 tillers after wind + brushing (Fig. 5D) with no significant difference between the two treatments.

Both outdoor treatments resulted in a significantly reduced flag leaf area of the main tiller. Based on flag leaf length and width, the flag leaf areas were calculated at the end of the treatment and at the end of flowering (Supplementary Data Table S6). Natural wind exposure reduced the leaf area at the end of treatment, though this was not significant, while a further and significant reduction was observed when wind exposure was supplemented with brushing (a reduction in leaf area by 34 % compared with control). These differences became more pronounced after flowering, with a significant reduction in leaf area for both treatments, a 25 % reduction for wind and 42 % reduction for wind + brushing.

**Fig. 6.**
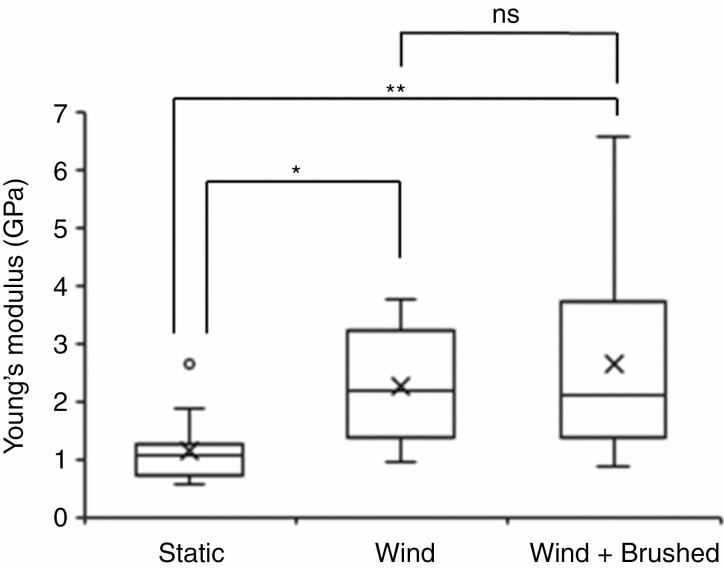
Both natural wind and wind supplemented with brushing trigger an increase in the Young’s modules of stem segments from senesced mature plants. Significant differences to untreated controls (Dunnett’s test) are indicated by asterisks (*P < 0.05, **P < 0.01, ***P < 0.001; ns, not significant). ANOVA with a post-hoc Tukey test was performed to identify statistical differences between wind and wind + brushed treatments (*P* ≤ 0.05).

Whole above-ground biomass measurements showed a decrease after both treatments (20 %), but these were not significant (Fig. 5E). Both treatments resulted in a marked and significant increase of the Young’s modulus of the third internode when compared to controls, almost doubling upon wind treatment with a further increase when natural exposure to wind was supplemented with brushing (Fig. 6).

In summary, these results showed that exposure to natural wind generally induces similar phenotypic effects to those seen in controlled environmental conditions.

### Effect of pseudo-natural conditions on spike and grain measures

Although 2-week-old plants showed a significant increase in number of spikes per plant upon brushing in the dose–response experiments, this was not the case in the outdoor experiment. The number of spikes per plant was increased for both treatments, but these increases were not significant. Other spike measures were also not significantly different upon the treatments, except for the average plant spike weight after natural wind exposure, which was significantly lower compared to controls (1.43 g vs. 1.72 g, respectively) (Table 3). Note that the number of spikes for the outdoor experiment was overall lower (all within an average of 3.0 to 3.5 spikes per plant, Table 3) when compared with both controlled environment experiments (ranging between 4.8 and 8.6 spikes per plant).

**Table 3.** Spike and grain measurements related to the outdoor experiment

		Treatment		
	Static	Wind	Brushed	Brushed vs. wind (Tukey HSD)
**T2 Spike count (*n* = 24)**				
	3.1 ± 0.17	3.4 ± 0.20	3.5 ± 0.22	
		*P* = 0.573	*P* = 0.385	*P* = 0.952
**T3 main tiller spike length (mm; *n* = 20)**				
	75.32 ± 1.789	71.93 ± 2.556	69.13 ± 2.012	
		*P* = 0.426	*P* = 0.066	*P* = 0.636
**T3 main tiller spike weight (g; *n* = 12)**				
	1.29 ± 0.169	1.14 ± 0.206	1.12 ± 0.164	
		*P* = 0.795	*P* = 0.734	*P* = 0.995
**T3 average spike length (mm; *n* = 12)**				
	79.70 ± 1.374	76.24 ± 2.104	73.85 ± 1.586	
		*P* = 0.275	*P* = 0.040	*P* = 0.593
			*	
	a	ab	b	
**T3 average spike weight (g; *n* = 12)**				
	1.72 ± 0.092	1.70 ± 0.094	1.43 ± 0.048	
		*P* = 0.975	*P* = 0.034	*P* = 0.071
			*	
	a	ab	b	
	Static	Wind	Brushed	
***n* = **	202	206	252	
**Grain length (mm)**				
	4.60 ± 0.035	4.51 ± 0.032	4.35 ± 0.030	
		*P* = 0.074	*P* < 0.001	*P* = 0.002
			***	
	a	a	b	
**Grain depth (mm)**				
	2.72 ± 0.027	2.65 ± 0.031	2.68 ± 0.024	
		*P* = 0.139	*P* = 0.565	*P* = 0.609
**Grain width (mm)**				
	3.58 ± 0.026	3.52 ± 0.028	3.37 ± 0.024	
		*P* = 0.188	*P* < 0.001	*P* < 0.001
			***	
	a	a	b	
**Mean grain volume (mm**^**3**^)				
	25.12 ± 0.389	23.54 ± 0.395	22.21 ± 0.336	
		*P* = 0.007	*P* < 0.001	*P* = 0.028
		**	***	
	a	b	c	
**Mean grain count per main tiller**				
	18.4 ± 3.63	17.2 ± 4.49	21.0 ± 3.91	
		*P* = 0.969	*P* = 0.859	*P* = 0.778
**Total grain volume (mm**^**3**^)				
	563.75 ± 66.847	538.73 ± 107.295	506.21 ± 82.342	
		*P* = 0.972	*P* = 0.849	*P* = 0.961

Significant differences to the static treatment are indicated by asterisks (*P < 0.05, **P < 0.01, ***P < 0.001).

ANOVA with a post-hoc Tukey test was performed to identify statistical differences (*P* < 0.05), indicated by lower case letters.

Data in this table are means ± standard error.

When looking at the grains, exposure to wind + brushing significantly decreased grain length and grain width (Table 3) while the average individual grain volume was significantly reduced for both treatments compared with controls, with the individual grain volume significantly reduced by wind + brushing compared with wind treatment only (Table 3). The total number of grains per main tiller spike was not affected (Table 3) and despite the significant reduction in individual grain volume for both wind-treated and wind + brushed plants, there was no significant difference in total grain volume per main tiller (Table 3).

## DISCUSSION

Although it is well known that wheat is sensitive to mechanical perturbation, most studies have focused on aspects related to lodging as this represents a major problem for cereal production, with severe lodging episodes having significant economic impact (Berry *et al.*, 2004; Berry and Spink, 2012). However, our knowledge on the effect of mild mechanical stimulation on wheat growth and development is surprisingly limited considering the importance of this crop to human civilization. A recent study in the model grass *Brachypodium distachyon* highlighted that exposure of grasses to moderate mechanical stimulation is a relevant environmental factor affecting multiple traits important for their utilization in food, feed and bioenergy applications (Gladala-Kostarz *et al.*, 2020). In this study, we evaluated the effect of mechanical stimulation in wheat and demonstrate that the morphological and developmental response to mechanical brushing treatment, both in relation to vegetative above-ground biomass, as well as responses associated with grain yield, is dependent on plant age as well as the dose of the treatments.

### Age– and dose–response relationships between mechanical stimulation and wheat growth and development

Using controlled brushing treatments as a surrogate for natural mechanical stimulation, our study presents, to our knowledge, the first detailed analyses of the multi-level effects of moderate mechanical stimulation on wheat growth and development.

It is well known that a plant’s response to environmental stresses is dependent on the intensity of stimulus and the developmental stage of the plant when the stimulus is received. Here we revealed a clear age–response effect, using 20 daily brushstrokes, with 2-week-old wheat plants showing a more profound phenotypic/morphometric response to mechanical stimulation compared to the 4- and 6-week-old age-groups. This was particularly reflected in the height measurements after the treatments, with an ~40 % reduction in plant height observed for the 2-week-old age-group. However, reductions in main tiller height, although significant, were not different between the age-groups when measured at the end of flowering, suggesting that age-dependent reductions in height at early developmental stages were offset by growth recovery mechanisms after the treatments. A gradual return to normal growth rates for most of the plant species evaluated was also observed by Jaffe (1973) when the mechanical stimulus was discontinued. This is corroborated by the internode length measurements, which showed a notable progression in internode response of each age-group to mechanical treatments, with the length of the fourth internode of the 2-week age-group not significantly different upon treatment compared with untreated controls, while this internode was shorter after treatment for the other two age-groups when compared with respective controls. Remarkably, dose–response measurements showed that plant height was already significantly reduced even with one daily brushstroke when compared with untreated plants with a possible saturation in the height reduction response from three daily brushstrokes onwards. Although reductions in plant height as a consequence of inhibition of stem elongation in response to mechanical treatments have been commonly observed in other studies (reviewed by Biddington, 1986 and Gardiner *et al.*, 2016), including for wheat (Crook and Ennos, 1996), this is to our knowledge the first report where the effect of applying mechanical treatment to wheat plants at different developmental stages and with different doses has been evaluated.

Most studies on thigmomorphogenesis in dicot species showed that mechanical stimulation results in a decrease of the Young’s modulus of stem segments, and hence a lower stiffness of the stem (Anten *et al.*, 2005; Paul-Victor and Rowe, 2011). However, mechanical stimulation in wheat resulted in an opposite effect, with an increase of the Young’s modulus of senesced stem segments compared with untreated controls. These findings are in agreement with those reported for maize stems (Goodman and Ennos, 1996) and recently also for *Brachypodium* stems (Gladala-Kostarz *et al.*, 2020), and suggest that the perception and transduction of mechanical stimuli can lead to the establishment of different mechanical properties of stem tissues in the monocot grasses (Poaceae) when compared to those in dicots. It remains to be seen if these differences are related to the distinct morphological and anatomical organization of the vasculature of the grasses, lacking a specialized cambium layer and therefore not undergoing secondary growth like most dicots (Handakumbura and Hazen, 2012).

Besides a reduction in plant height, an increase in tiller numbers of the youngest age-group was the most obvious phenotypic response to mechanical stimulation. Results from the dose–response experiment showed that even one daily brushstroke increased the tiller number to a similar extent as 20 brushstrokes. Tillering capacity is one of the most important agronomic traits in wheat as it determines the number of spikes per plant, thus affecting grain yield (Naruoka *et al.*, 2011; Xu *et al.*, 2017). Increases in tiller number as a result of mechanical stimulation were also found in rice (*Oryza sativa* L.) (Zhao *et al.*, 2018). However, tiller numbers were unaffected by mechanical stimulation in *Brachypodium* (Gladala-Kostarz *et al.*, 2020). Since the tiller number of wheat is controlled by the environment at early developmental stages, from the three-leaf stage (DGS 13) up to the beginning of stem elongation (DGS 30) (Tottman, 1987; Tilley *et al.*, 2019), the timing of mechanical stimulation plays an important role in affecting tiller numbers. Indeed, tiller numbers were unaffected when 4- and 6-week-old wheat plants, starting at DGS ~37–39 and DGS ~51, respectively, were exposed to mechanical stimulation.

The increase in tiller number and reduction in height observed after mechanical stimulation are in agreement with the reported negative correlation between tiller number and height in wheat (Xu *et al.*, 2017). Height reductions in cereals are often associated with changes in the synthesis of the hormones gibberellin and brassinosteroids or the pathways they act upon (Wang and Li, 2008), and it will be interesting to determine if height reductions induced by mechanical stimulation are similarly related to such hormonal actions. Lateral bud outgrowth is often supressed by apical dominance and variations in tiller number have been mapped to various quantitative trait loci (Xu *et al.*, 2017). Although several genetic components involved in branching, including the carotenoid-derived long-range signalling pathway involving MAX genes, have been confirmed to operate in the monocot grasses (Zou *et al.*, 2006; Arite *et al.*, 2007; Wang and Li, 2008) the ability to induce increases in tillering through mechanical stimulation may provide a route to improve our knowledge of the molecular mechanisms involved in controlling tillering in wheat. Linked to the increase in tiller number, it is interesting to note that mechanical stimulation also resulted in increased above-ground biomass measures of senesced mature plants for the youngest age group, despite the decrease in overall stem length.

### Grain characteristics in wheat are affected by mechanical stimulation

Grain yield is the most important agronomic trait of wheat and it is known that stressful environmental conditions such as drought, temperature, nutrient limitations and waterlogging can affect grain yield and quality. Prior to anthesis, grain number is impacted by the effect of environmental conditions on photosynthesis, tiller formation and the development of the inflorescence, while post-anthesis, environmental conditions will predominantly affect grain size and composition (Dupont and Altenbach, 2003). Although reports have shown that windbreaks and shelters can lead to yield increases for cereals, including wheat (Brandle *et al.*, 2009), here we present, to our knowledge, the first detailed assessment of the effect that mechanical stimulation can have on grain characteristics in wheat. Our results showed that spike and grain development were particularly affected when young wheat plants, containing two to three tillers (DGS 22–23) were exposed to mechanical stimulation for a 4-week period until the emergence of the flag leaf (DGS 37–39). Although mechanical stimulation of these young wheat seedlings led to an increase in tillers, total spike weight per plant was not affected while the average spike weight was significantly reduced. Analysis of µCT images showed that the concomitant reductions in the number of grains and average volume of individual grains led to a 4-fold reduction of total grain volume of the main tiller spike. Remarkably, these treatment-induced changes in grain characteristics were not significantly different between the doses of brushing applied, with one daily brushstroke having a similar effect to 20 daily brushstrokes. These results suggest that although wheat plants grown under still conditions are capable of achieving higher grain yields, such conditions are completely artificial with plants out in the field being constantly exposed to varying levels of mechanical stimulation. Nevertheless, the effect on grain number and volume upon mechanical treatment of the youngest age-group, with treatments occurring well before grain filling and even before the onset of anthesis, raises the question of how mechanical stimulation at such early developmental stages has such a dramatic effect. By contrast, treatment of both older age groups, which overlapped with grain formation and filling, did not lead to large changes in morphological grain characteristics. Floret initiation and development starts at the early stages of the stem elongation phase (Guo and Schnurbusch, 2015; Guo *et al.*, 2018), DGS 30–39, which overlaps with the treatment period of the youngest age group. Drought and nutritional stress are known to affect floret fertility, therefore reducing grain numbers (Barnabás *et al.*, 2008). The observed changes in grain characteristics may therefore be a direct consequence of a mechano-perception signalling cascade triggered by the brushing treatments. Grain numbers are highly dependent on the environmental conditions present prior to and during formation of the flower (Sinclair and Jamieson, 2006), with the most dramatic effects on yield observed when stress coincides with early stages of meiosis through to early grain initiation (Saini, 1997; Barnabás *et al.*, 2008). Alternatively, the increased tiller numbers induced by mechanical stimulation may increase the competition with the main shoot for resources (Foulkes *et al*., 2011). It has been shown that the removal of tillers in wheat plants increases grain numbers (Kemp and Whingwiri, 1980) and grain size (Gu and Marshall, 1988). However, mechanical stimulation reduced seed numbers and yield in *Brachypodium*, while tiller numbers were not affected (Gladala-Kostarz *et al.*, 2020). A better understanding of the underlying developmental and resource-allocation-dependent factors is required to establish a mechanistic link between mechanical stimulation and grain yields.

### Controlled environment vs. outdoor conditions

The controlled environment experiments, discussed so far, allowed us to study the effects of applying defined treatments to wheat plants. It is important to acknowledge that such controlled conditions do not reflect those of the natural environment. To a large extent, the outdoor experiment, with treatments of the 2-week age-group, confirmed the phenotypic alterations observed by mechanical stimulation of the same age-group in the controlled environment experiments, reducing height, increasing tiller number and increasing the Young’s modulus of stem segments. However, in contrast to the controlled environment experiments, the effects seen on spike and grain measures were less dramatic for the outdoor experiment. Although outdoor treatments increased the numbers of spikes per plant, the absolute numbers were about half of those for the controlled environment experiments and also about half of the corresponding tiller numbers. The latter indicates that at least 50 % of the tillers did not survive to the point of spike production despite the fact that tiller numbers showed similar treatment-induced increases as observed for the controlled environment experiments. It is known that 10–80 % of the tillers initiated in wheat can be aborted before anthesis and this is affected by environmental conditions (Xie *et al.*, 2016; Tilley *et al.*, 2019). During the outdoor pot experiment, wheat plants may have experienced a period of water and/or heat stress as plants were exposed to a period of warm and dry weather. Nevertheless, albeit not always significant, the direction of the changes in the measurements for spike- and grain-related traits induced by mechanical stimulation were mostly the same as observed under controlled environment conditions, with a significant reduction in individual grain volume. Note that the natural exposure to wind, supplemented with daily brushing, had a significant effect on grain dimensions (length, width and volume) when compared to natural wind only, suggesting that the response to mechanical stimulation, at least regarding these measures, was not saturated.

### Conclusions and future perspectives

This study revealed a remarkable age– and dose–response of wheat to mechanical stimulation. Besides affecting plant phenological traits, the treatment of 2-week-old wheat plants significantly affected spike and grain development. Together with the results from outdoor experiments, the outcomes highlight the complexity of the response to mechanical stimulation. Further studies are required to characterize the implications of mechanical stimulation, to evaluate the genotypic diversity in the responses, and to identify and dissect the molecular mechanisms involved in the perception and transduction of mechanical stimuli that lead to the observed morphogenetic responses. It will also be important to evaluate the interaction between genotypes and mechanical stimulation on grain quality measures as well as on processability parameters. While excess mechano-stimulation can clearly be harmful, traditional Japanese farmers have applied mechanical stimulation for centuries to wheat seedlings by trampling on them, a process called ‘mugifumi’, in order to make them more resilient and improve yields (Iida, 2014). Although there is little knowledge on the dose–response nor the molecular mechanisms underlying mechano-sensing and mechano-transduction in cereals, our results showing that mechanical stimulation can increase above-ground biomass, increase tiller numbers and affect grain development, corroborated by the tradition of ‘mugifumi’, highlight the importance of mechanical stimulation for wheat productivity. A better understanding of thigmomorphogenesis in wheat could lead to mechanical conditioning treatments in agriculture that would provide an environmentally friendly alternative to the utilization of plant growth regulators.

## SUPPLEMENTARY DATA

Supplementary data are available online at https://academic.oup.com/aob and consist of the following. Movie S1: experimental set-up of the controlled mechanical stimulation treatments. Table S1: wind and brushing trigger similar phenotypic responses in wheat. Table S2: the effect of brushing on the size of the main tiller flag leaf is age-dependent. Table S3: brushing induces age-dependent reductions in internode lengths. Table S4: brushing 2-week-old plants increases Young’s modulus. Table S5: dose–response effects of brushing treatments on internode length and diameter. Table S6: additional phenotypic measurements related to stem height and leaf size in response to outdoor treatments. Table S7: internode length and diameter measurements following the outdoor experiment. Figure S1: experimental set-up for the outdoor experiment. Methods S1: determination of below-ground dry biomass. Methods S2: equations used for calculating Young’s modulus.

mcab070_suppl_Supplementary_Video_S1

mcab070_suppl_Supplementary_Video_S2

mcab070_suppl_Supplementary_Material
