# Supplementary material for: Mechanical stimulation in wheat triggers age- and dose-dependent alterations in growth, development and grain characteristics
Source: Ann Bot. 2021 Jun 6;128(5):589–603. doi: 10.1093/aob/mcab070 (PMC8422892; doi:10.1093/aob/mcab070)

## Slide 1
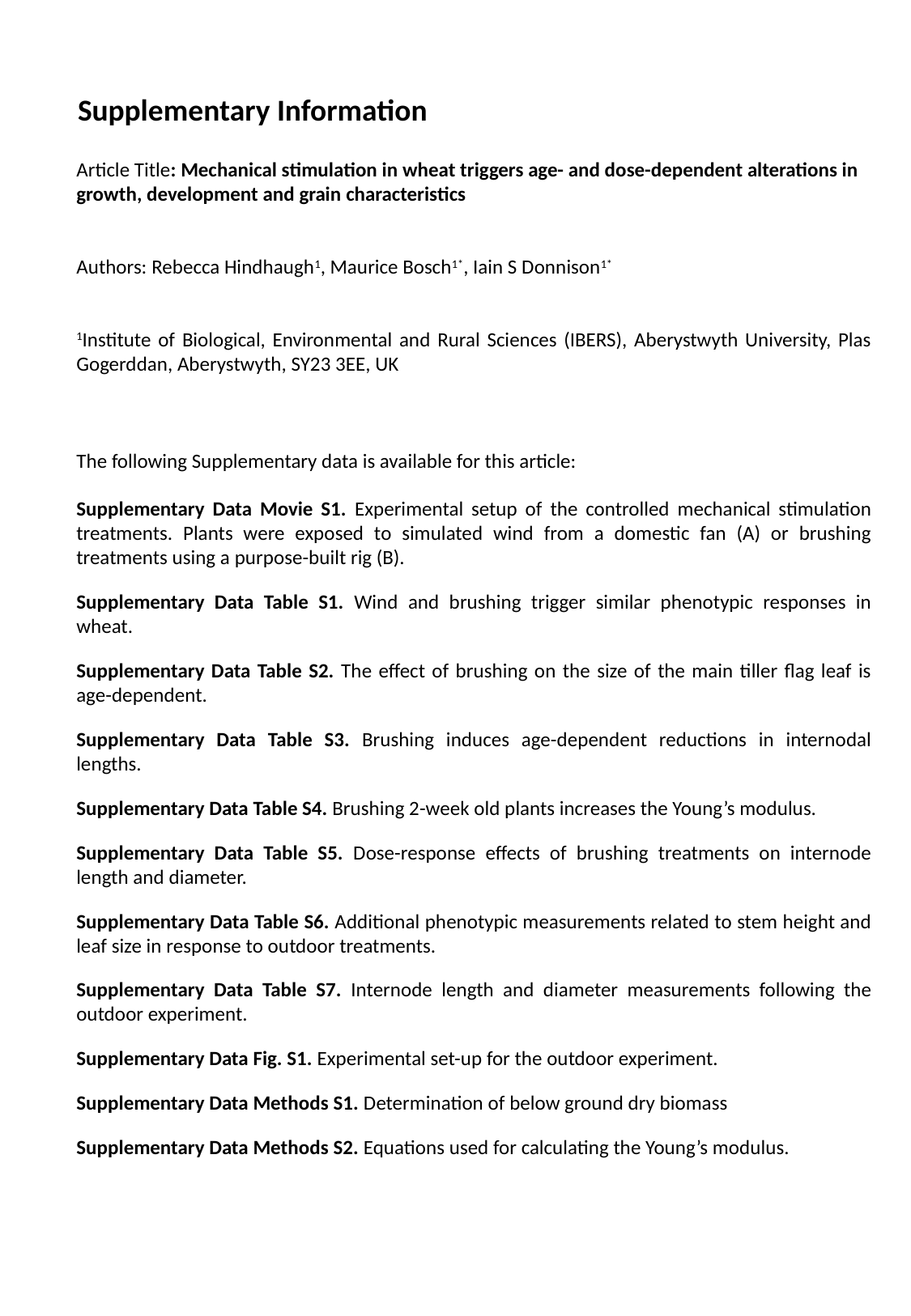

Supplementary Information
Article Title: Mechanical stimulation in wheat triggers age- and dose-dependent alterations in growth, development and grain characteristics
Authors: Rebecca Hindhaugh1, Maurice Bosch1*, Iain S Donnison1*
1Institute of Biological, Environmental and Rural Sciences (IBERS), Aberystwyth University, Plas Gogerddan, Aberystwyth, SY23 3EE, UK
The following Supplementary data is available for this article:
Supplementary Data Movie S1. Experimental setup of the controlled mechanical stimulation treatments. Plants were exposed to simulated wind from a domestic fan (A) or brushing treatments using a purpose-built rig (B).
Supplementary Data Table S1. Wind and brushing trigger similar phenotypic responses in wheat.
Supplementary Data Table S2. The effect of brushing on the size of the main tiller flag leaf is age-dependent.
Supplementary Data Table S3. Brushing induces age-dependent reductions in internodal lengths.
Supplementary Data Table S4. Brushing 2-week old plants increases the Young’s modulus.
Supplementary Data Table S5. Dose-response effects of brushing treatments on internode length and diameter.
Supplementary Data Table S6. Additional phenotypic measurements related to stem height and leaf size in response to outdoor treatments.
Supplementary Data Table S7. Internode length and diameter measurements following the outdoor experiment.
Supplementary Data Fig. S1. Experimental set-up for the outdoor experiment.
Supplementary Data Methods S1. Determination of below ground dry biomass
Supplementary Data Methods S2. Equations used for calculating the Young’s modulus.

## Slide 2
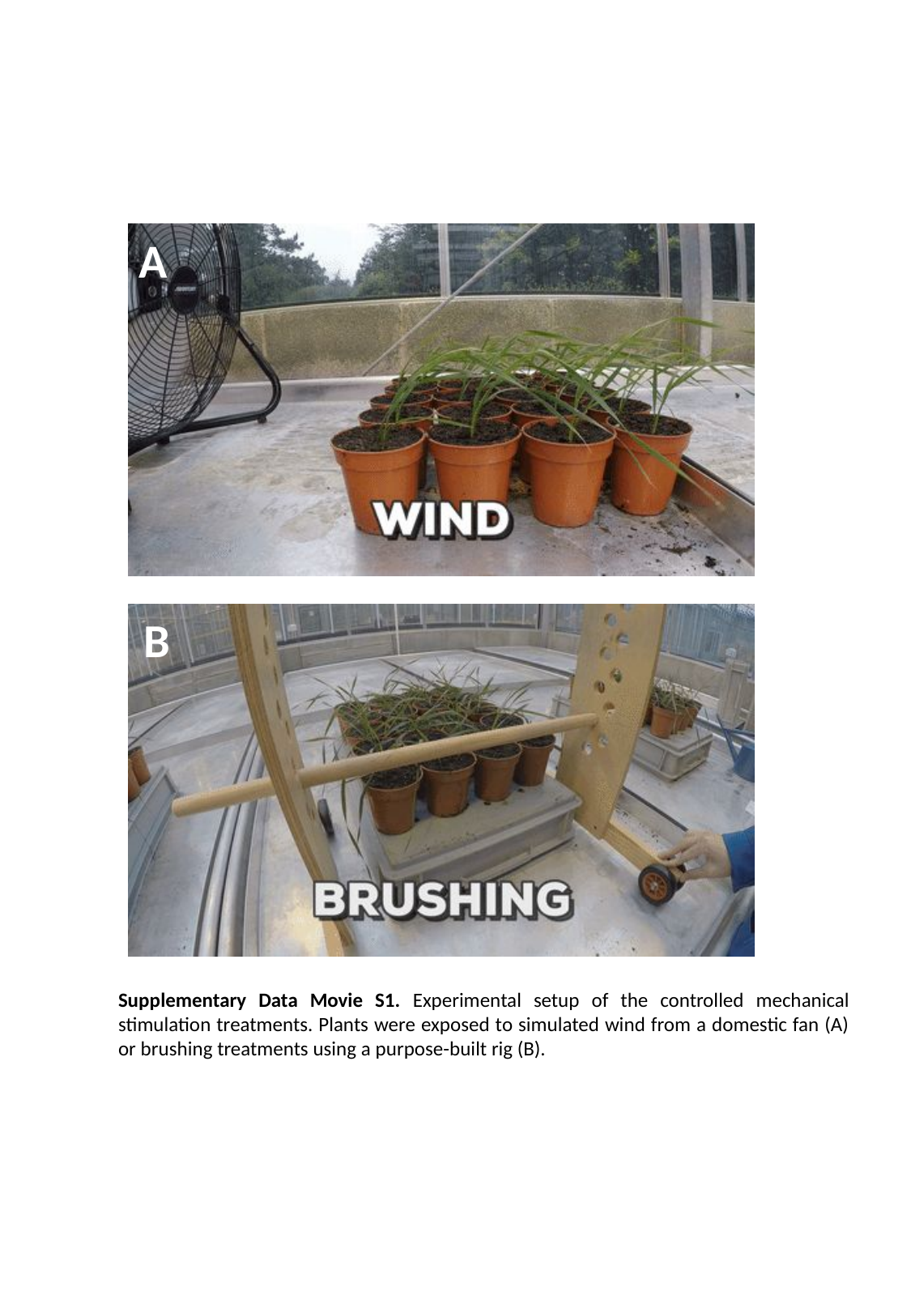

A
B
Supplementary Data Movie S1. Experimental setup of the controlled mechanical stimulation treatments. Plants were exposed to simulated wind from a domestic fan (A) or brushing treatments using a purpose-built rig (B).

## Slide 3
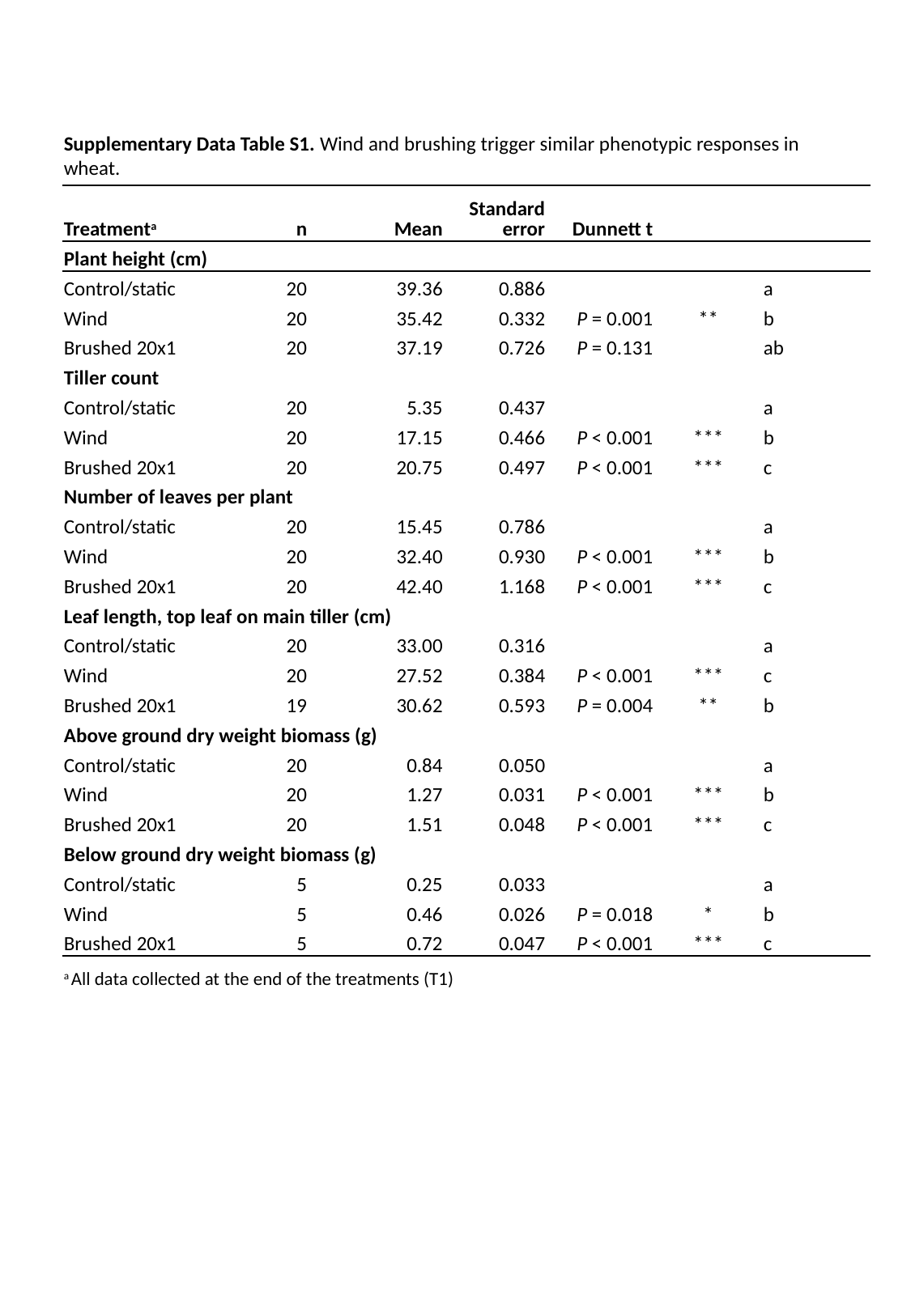

Supplementary Data Table S1. Wind and brushing trigger similar phenotypic responses in wheat.
| Treatmenta | n | n | Mean | Standard error | Dunnett t | | |
| --- | --- | --- | --- | --- | --- | --- | --- |
| Plant height (cm) | | | | | | | |
| Control/static | 20 | 20 | 39.36 | 0.886 | | | a |
| Wind | 20 | 20 | 35.42 | 0.332 | P = 0.001 | \*\* | b |
| Brushed 20x1 | 20 | 20 | 37.19 | 0.726 | P = 0.131 | | ab |
| Tiller count | | | | | | | |
| Control/static | 20 | 20 | 5.35 | 0.437 | | | a |
| Wind | 20 | 20 | 17.15 | 0.466 | P < 0.001 | \*\*\* | b |
| Brushed 20x1 | 20 | 20 | 20.75 | 0.497 | P < 0.001 | \*\*\* | c |
| Number of leaves per plant | | | | | | | |
| Control/static | 20 | | 15.45 | 0.786 | | | a |
| Wind | 20 | | 32.40 | 0.930 | P < 0.001 | \*\*\* | b |
| Brushed 20x1 | 20 | | 42.40 | 1.168 | P < 0.001 | \*\*\* | c |
| Leaf length, top leaf on main tiller (cm) | | | | | | | |
| Control/static | 20 | | 33.00 | 0.316 | | | a |
| Wind | 20 | | 27.52 | 0.384 | P < 0.001 | \*\*\* | c |
| Brushed 20x1 | 19 | | 30.62 | 0.593 | P = 0.004 | \*\* | b |
| Above ground dry weight biomass (g) | | | | | | | |
| Control/static | 20 | | 0.84 | 0.050 | | | a |
| Wind | 20 | | 1.27 | 0.031 | P < 0.001 | \*\*\* | b |
| Brushed 20x1 | 20 | | 1.51 | 0.048 | P < 0.001 | \*\*\* | c |
| Below ground dry weight biomass (g) | | | | | | | |
| Control/static | 5 | | 0.25 | 0.033 | | | a |
| Wind | 5 | | 0.46 | 0.026 | P = 0.018 | \* | b |
| Brushed 20x1 | 5 | | 0.72 | 0.047 | P < 0.001 | \*\*\* | c |
a All data collected at the end of the treatments (T1)

## Slide 4
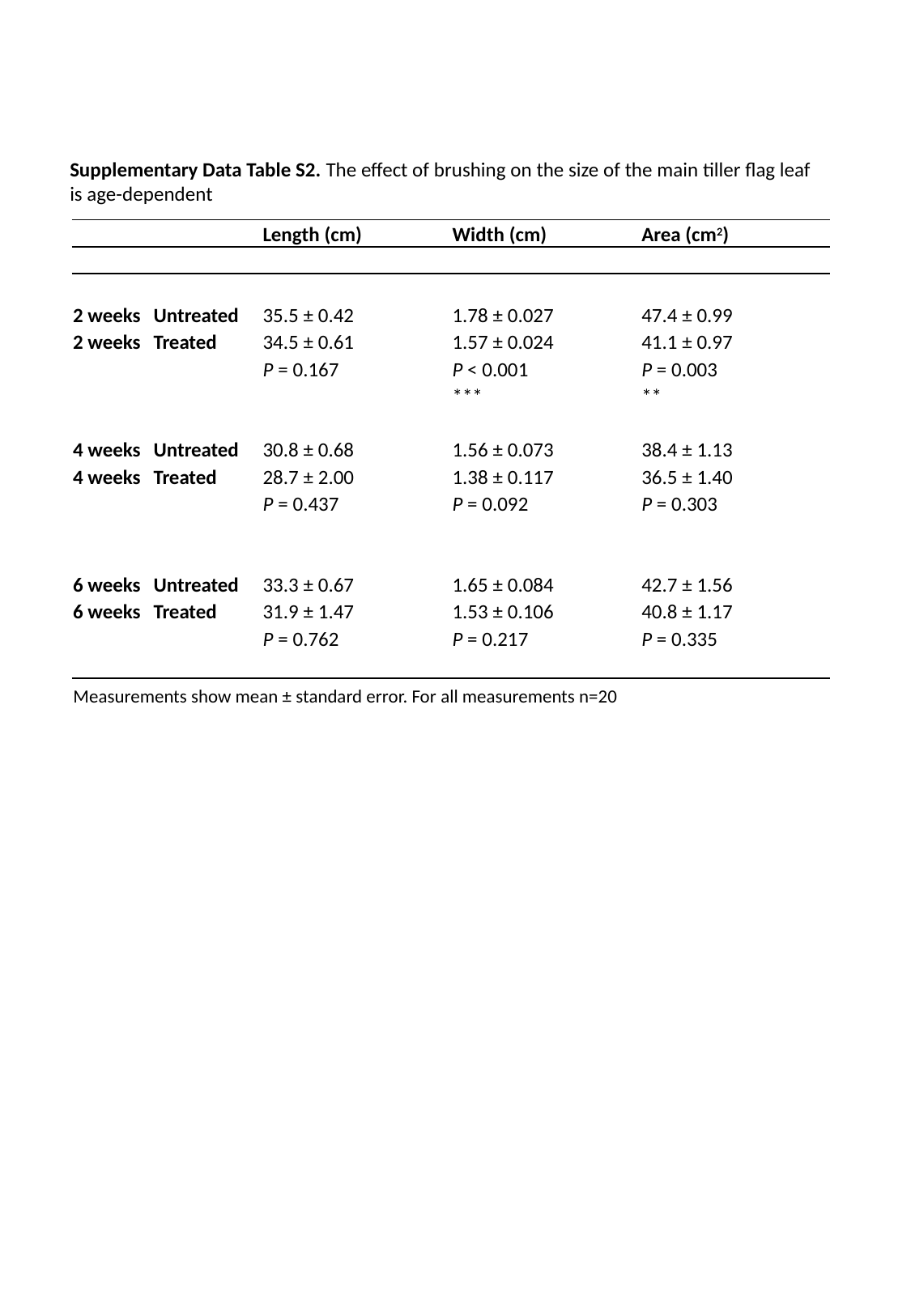

Supplementary Data Table S2. The effect of brushing on the size of the main tiller flag leaf is age-dependent
| | | Length (cm) | Width (cm) | Area (cm2) |
| --- | --- | --- | --- | --- |
| | | | | |
| | | | | |
| 2 weeks | Untreated | 35.5 ± 0.42 | 1.78 ± 0.027 | 47.4 ± 0.99 |
| 2 weeks | Treated | 34.5 ± 0.61 | 1.57 ± 0.024 | 41.1 ± 0.97 |
| | | P = 0.167 | P < 0.001 | P = 0.003 |
| | | | \*\*\* | \*\* |
| | | | | |
| 4 weeks | Untreated | 30.8 ± 0.68 | 1.56 ± 0.073 | 38.4 ± 1.13 |
| 4 weeks | Treated | 28.7 ± 2.00 | 1.38 ± 0.117 | 36.5 ± 1.40 |
| | | P = 0.437 | P = 0.092 | P = 0.303 |
| | | | | |
| | | | | |
| 6 weeks | Untreated | 33.3 ± 0.67 | 1.65 ± 0.084 | 42.7 ± 1.56 |
| 6 weeks | Treated | 31.9 ± 1.47 | 1.53 ± 0.106 | 40.8 ± 1.17 |
| | | P = 0.762 | P = 0.217 | P = 0.335 |
| | | | | |
Measurements show mean ± standard error. For all measurements n=20

## Slide 5
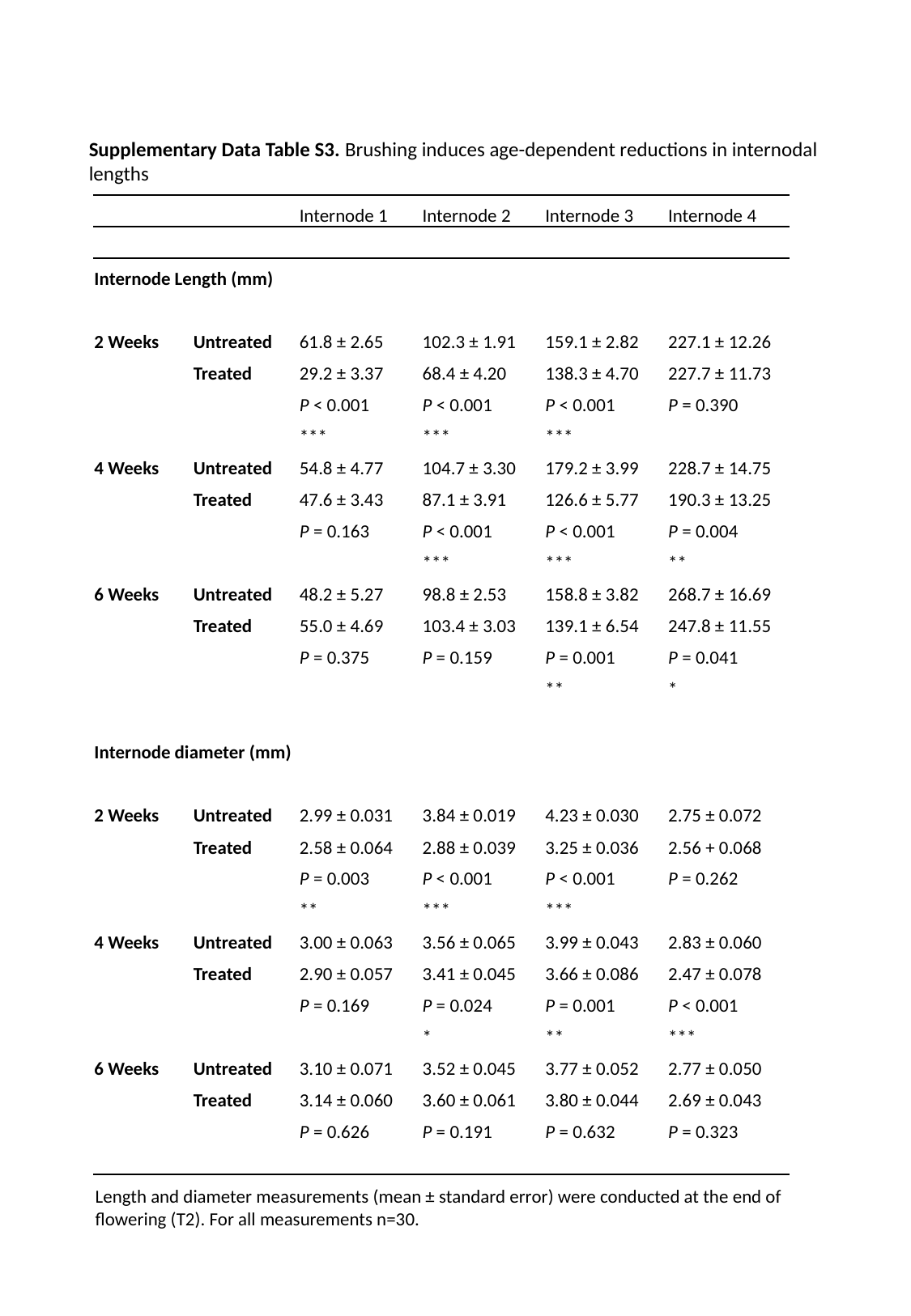

Supplementary Data Table S3. Brushing induces age-dependent reductions in internodal lengths
| | | Internode 1 | Internode 2 | Internode 3 | Internode 4 |
| --- | --- | --- | --- | --- | --- |
| | | | | | |
| Internode Length (mm) | | | | | |
| | | | | | |
| 2 Weeks | Untreated | 61.8 ± 2.65 | 102.3 ± 1.91 | 159.1 ± 2.82 | 227.1 ± 12.26 |
| | Treated | 29.2 ± 3.37 | 68.4 ± 4.20 | 138.3 ± 4.70 | 227.7 ± 11.73 |
| | | P < 0.001 | P < 0.001 | P < 0.001 | P = 0.390 |
| | | \*\*\* | \*\*\* | \*\*\* | |
| 4 Weeks | Untreated | 54.8 ± 4.77 | 104.7 ± 3.30 | 179.2 ± 3.99 | 228.7 ± 14.75 |
| | Treated | 47.6 ± 3.43 | 87.1 ± 3.91 | 126.6 ± 5.77 | 190.3 ± 13.25 |
| | | P = 0.163 | P < 0.001 | P < 0.001 | P = 0.004 |
| | | | \*\*\* | \*\*\* | \*\* |
| 6 Weeks | Untreated | 48.2 ± 5.27 | 98.8 ± 2.53 | 158.8 ± 3.82 | 268.7 ± 16.69 |
| | Treated | 55.0 ± 4.69 | 103.4 ± 3.03 | 139.1 ± 6.54 | 247.8 ± 11.55 |
| | | P = 0.375 | P = 0.159 | P = 0.001 | P = 0.041 |
| | | | | \*\* | \* |
| | | | | | |
| Internode diameter (mm) | | | | | |
| | | | | | |
| 2 Weeks | Untreated | 2.99 ± 0.031 | 3.84 ± 0.019 | 4.23 ± 0.030 | 2.75 ± 0.072 |
| | Treated | 2.58 ± 0.064 | 2.88 ± 0.039 | 3.25 ± 0.036 | 2.56 + 0.068 |
| | | P = 0.003 | P < 0.001 | P < 0.001 | P = 0.262 |
| | | \*\* | \*\*\* | \*\*\* | |
| 4 Weeks | Untreated | 3.00 ± 0.063 | 3.56 ± 0.065 | 3.99 ± 0.043 | 2.83 ± 0.060 |
| | Treated | 2.90 ± 0.057 | 3.41 ± 0.045 | 3.66 ± 0.086 | 2.47 ± 0.078 |
| | | P = 0.169 | P = 0.024 | P = 0.001 | P < 0.001 |
| | | | \* | \*\* | \*\*\* |
| 6 Weeks | Untreated | 3.10 ± 0.071 | 3.52 ± 0.045 | 3.77 ± 0.052 | 2.77 ± 0.050 |
| | Treated | 3.14 ± 0.060 | 3.60 ± 0.061 | 3.80 ± 0.044 | 2.69 ± 0.043 |
| | | P = 0.626 | P = 0.191 | P = 0.632 | P = 0.323 |
| | | | | | |
Length and diameter measurements (mean ± standard error) were conducted at the end of flowering (T2). For all measurements n=30.

## Slide 6
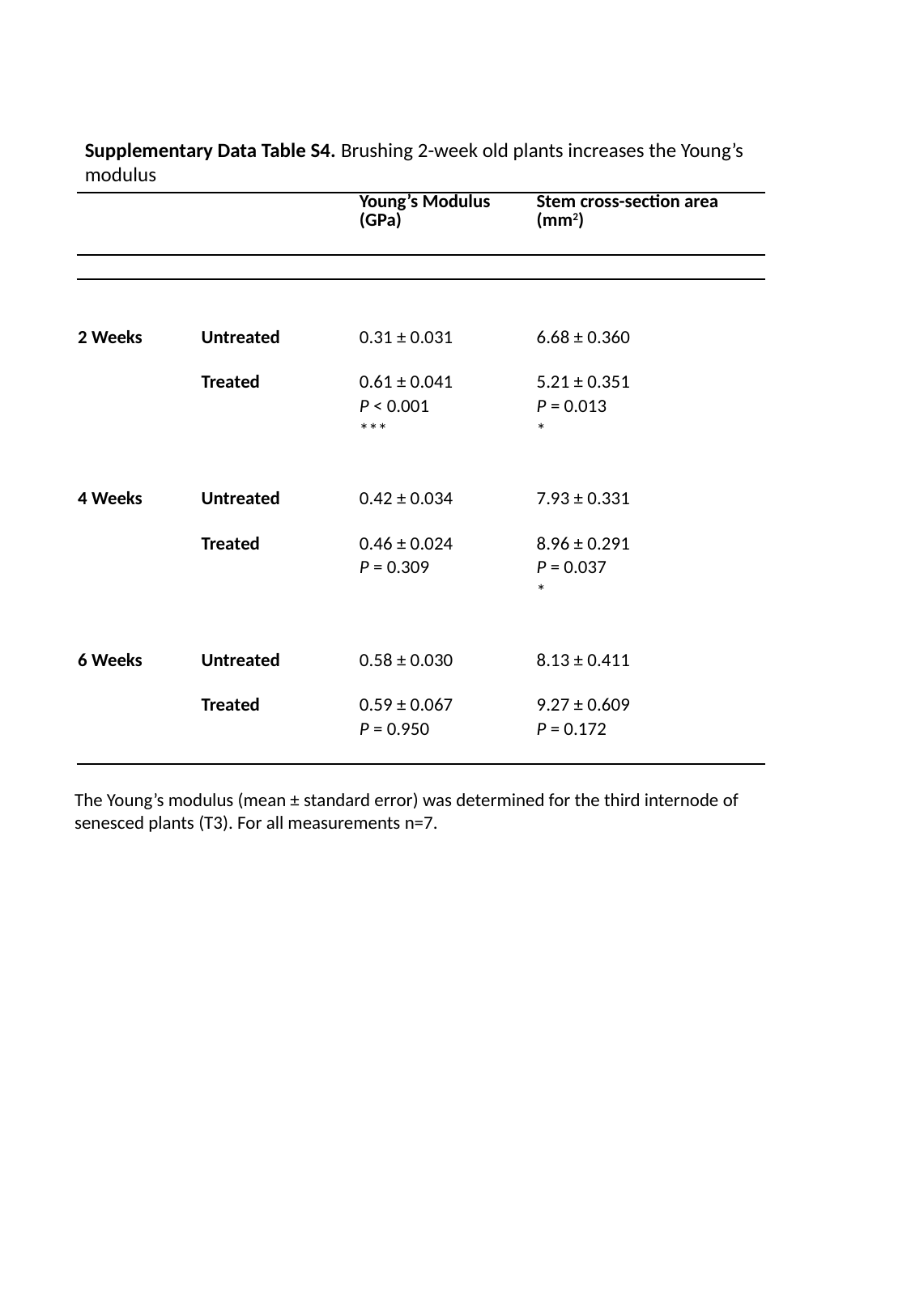

Supplementary Data Table S4. Brushing 2-week old plants increases the Young’s modulus
| | | Young’s Modulus (GPa) | Stem cross-section area (mm2) |
| --- | --- | --- | --- |
| | | | |
| | | | |
| | | | |
| 2 Weeks | Untreated | 0.31 ± 0.031 | 6.68 ± 0.360 |
| | Treated | 0.61 ± 0.041 | 5.21 ± 0.351 |
| | | P < 0.001 | P = 0.013 |
| | | \*\*\* | \* |
| | | | |
| 4 Weeks | Untreated | 0.42 ± 0.034 | 7.93 ± 0.331 |
| | Treated | 0.46 ± 0.024 | 8.96 ± 0.291 |
| | | P = 0.309 | P = 0.037 |
| | | | \* |
| | | | |
| 6 Weeks | Untreated | 0.58 ± 0.030 | 8.13 ± 0.411 |
| | Treated | 0.59 ± 0.067 | 9.27 ± 0.609 |
| | | P = 0.950 | P = 0.172 |
| | | | |
The Young’s modulus (mean ± standard error) was determined for the third internode of senesced plants (T3). For all measurements n=7.

## Slide 7
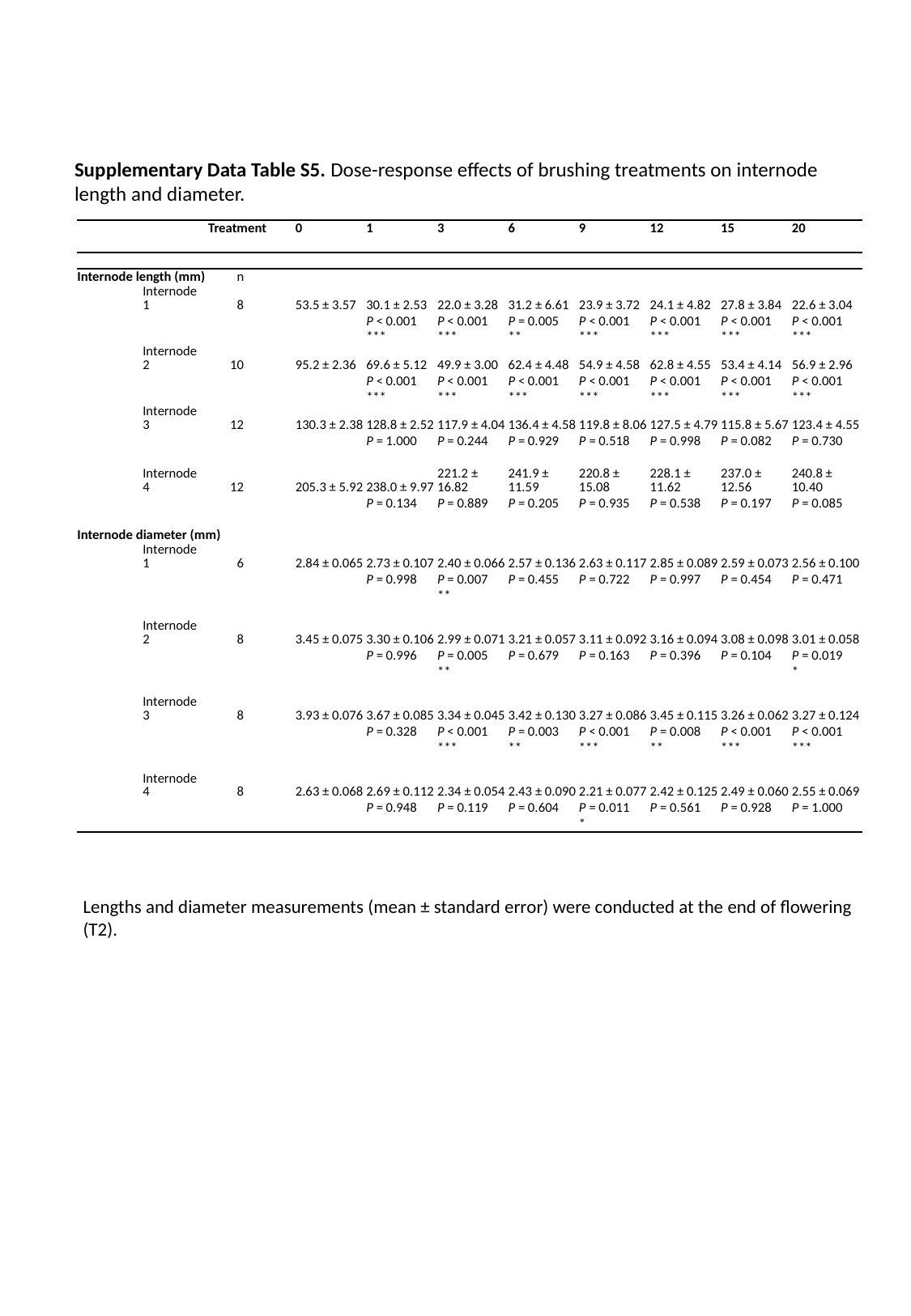

Supplementary Data Table S5. Dose-response effects of brushing treatments on internode length and diameter.
| | | Treatment | | 0 | 1 | 3 | 6 | 9 | 12 | 15 | 20 |
| --- | --- | --- | --- | --- | --- | --- | --- | --- | --- | --- | --- |
| | | | | | | | | | | | |
| | | | | | | | | | | | |
| Internode length (mm) | | n | | | | | | | | | |
| | Internode 1 | 8 | | 53.5 ± 3.57 | 30.1 ± 2.53 | 22.0 ± 3.28 | 31.2 ± 6.61 | 23.9 ± 3.72 | 24.1 ± 4.82 | 27.8 ± 3.84 | 22.6 ± 3.04 |
| | | | | | P < 0.001 | P < 0.001 | P = 0.005 | P < 0.001 | P < 0.001 | P < 0.001 | P < 0.001 |
| | | | | | \*\*\* | \*\*\* | \*\* | \*\*\* | \*\*\* | \*\*\* | \*\*\* |
| | Internode 2 | 10 | | 95.2 ± 2.36 | 69.6 ± 5.12 | 49.9 ± 3.00 | 62.4 ± 4.48 | 54.9 ± 4.58 | 62.8 ± 4.55 | 53.4 ± 4.14 | 56.9 ± 2.96 |
| | | | | | P < 0.001 | P < 0.001 | P < 0.001 | P < 0.001 | P < 0.001 | P < 0.001 | P < 0.001 |
| | | | | | \*\*\* | \*\*\* | \*\*\* | \*\*\* | \*\*\* | \*\*\* | \*\*\* |
| | Internode 3 | 12 | | 130.3 ± 2.38 | 128.8 ± 2.52 | 117.9 ± 4.04 | 136.4 ± 4.58 | 119.8 ± 8.06 | 127.5 ± 4.79 | 115.8 ± 5.67 | 123.4 ± 4.55 |
| | | | | | P = 1.000 | P = 0.244 | P = 0.929 | P = 0.518 | P = 0.998 | P = 0.082 | P = 0.730 |
| | | | | | | | | | | | |
| | Internode 4 | 12 | | 205.3 ± 5.92 | 238.0 ± 9.97 | 221.2 ± 16.82 | 241.9 ± 11.59 | 220.8 ± 15.08 | 228.1 ± 11.62 | 237.0 ± 12.56 | 240.8 ± 10.40 |
| | | | | | P = 0.134 | P = 0.889 | P = 0.205 | P = 0.935 | P = 0.538 | P = 0.197 | P = 0.085 |
| | | | | | | | | | | | |
| Internode diameter (mm) | | | | | | | | | | | |
| | Internode 1 | 6 | | 2.84 ± 0.065 | 2.73 ± 0.107 | 2.40 ± 0.066 | 2.57 ± 0.136 | 2.63 ± 0.117 | 2.85 ± 0.089 | 2.59 ± 0.073 | 2.56 ± 0.100 |
| | | | | | P = 0.998 | P = 0.007 | P = 0.455 | P = 0.722 | P = 0.997 | P = 0.454 | P = 0.471 |
| | | | | | | \*\* | | | | | |
| | | | | | | | | | | | |
| | Internode 2 | 8 | | 3.45 ± 0.075 | 3.30 ± 0.106 | 2.99 ± 0.071 | 3.21 ± 0.057 | 3.11 ± 0.092 | 3.16 ± 0.094 | 3.08 ± 0.098 | 3.01 ± 0.058 |
| | | | | | P = 0.996 | P = 0.005 | P = 0.679 | P = 0.163 | P = 0.396 | P = 0.104 | P = 0.019 |
| | | | | | | \*\* | | | | | \* |
| | | | | | | | | | | | |
| | Internode 3 | 8 | | 3.93 ± 0.076 | 3.67 ± 0.085 | 3.34 ± 0.045 | 3.42 ± 0.130 | 3.27 ± 0.086 | 3.45 ± 0.115 | 3.26 ± 0.062 | 3.27 ± 0.124 |
| | | | | | P = 0.328 | P < 0.001 | P = 0.003 | P < 0.001 | P = 0.008 | P < 0.001 | P < 0.001 |
| | | | | | | \*\*\* | \*\* | \*\*\* | \*\* | \*\*\* | \*\*\* |
| | | | | | | | | | | | |
| | Internode 4 | 8 | | 2.63 ± 0.068 | 2.69 ± 0.112 | 2.34 ± 0.054 | 2.43 ± 0.090 | 2.21 ± 0.077 | 2.42 ± 0.125 | 2.49 ± 0.060 | 2.55 ± 0.069 |
| | | | | | P = 0.948 | P = 0.119 | P = 0.604 | P = 0.011 | P = 0.561 | P = 0.928 | P = 1.000 |
| | | | | | | | | \* | | | |
Lengths and diameter measurements (mean ± standard error) were conducted at the end of flowering (T2).

## Slide 8
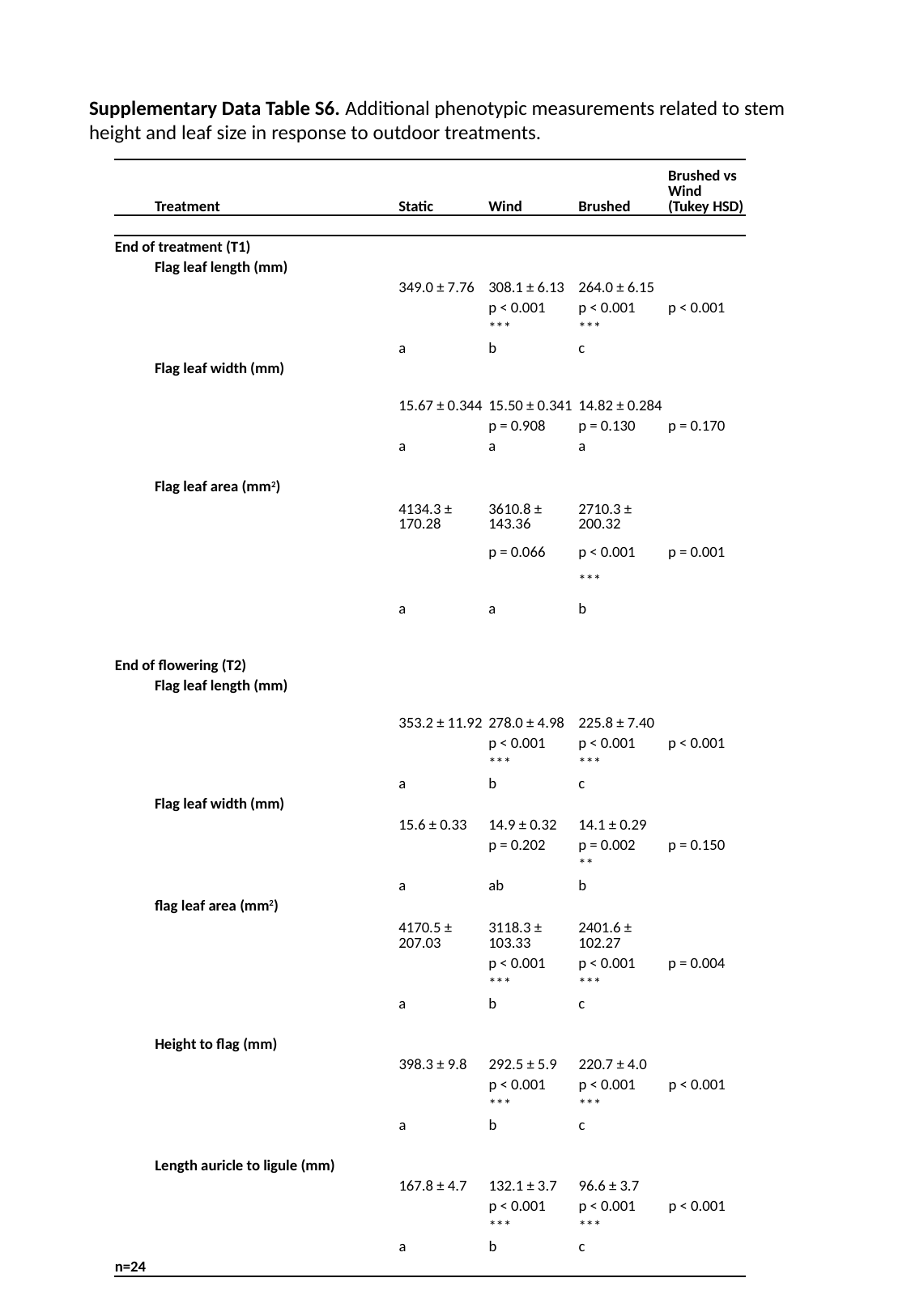

Supplementary Data Table S6. Additional phenotypic measurements related to stem height and leaf size in response to outdoor treatments.
| | Treatment | | | | | Static | Wind | Brushed | Brushed vs Wind (Tukey HSD) |
| --- | --- | --- | --- | --- | --- | --- | --- | --- | --- |
| | | | | | | | | | |
| End of treatment (T1) | | | | | | | | | |
| | Flag leaf length (mm) | | | | | | | | |
| | | | | | | 349.0 ± 7.76 | 308.1 ± 6.13 | 264.0 ± 6.15 | |
| | | | | | | | p < 0.001 | p < 0.001 | p < 0.001 |
| | | | | | | | \*\*\* | \*\*\* | |
| | | | | | | a | b | c | |
| | Flag leaf width (mm) | | | | | | | | |
| | | | | | | 15.67 ± 0.344 | 15.50 ± 0.341 | 14.82 ± 0.284 | |
| | | | | | | | p = 0.908 | p = 0.130 | p = 0.170 |
| | | | | | | a | a | a | |
| | | | | | | | | | |
| | Flag leaf area (mm2) | | | | | | | | |
| | | | | | | 4134.3 ± 170.28 | 3610.8 ± 143.36 | 2710.3 ± 200.32 | |
| | | | | | | | p = 0.066 | p < 0.001 | p = 0.001 |
| | | | | | | | | \*\*\* | |
| | | | | | | a | a | b | |
| | | | | | | | | | |
| End of flowering (T2) | | | | | | | | | |
| | Flag leaf length (mm) | | | | | | | | |
| | | | | | | 353.2 ± 11.92 | 278.0 ± 4.98 | 225.8 ± 7.40 | |
| | | | | | | | p < 0.001 | p < 0.001 | p < 0.001 |
| | | | | | | | \*\*\* | \*\*\* | |
| | | | | | | a | b | c | |
| | Flag leaf width (mm) | | | | | | | | |
| | | | | | | 15.6 ± 0.33 | 14.9 ± 0.32 | 14.1 ± 0.29 | |
| | | | | | | | p = 0.202 | p = 0.002 | p = 0.150 |
| | | | | | | | | \*\* | |
| | | | | | | a | ab | b | |
| | flag leaf area (mm2) | | | | | | | | |
| | | | | | | 4170.5 ± 207.03 | 3118.3 ± 103.33 | 2401.6 ± 102.27 | |
| | | | | | | | p < 0.001 | p < 0.001 | p = 0.004 |
| | | | | | | | \*\*\* | \*\*\* | |
| | | | | | | a | b | c | |
| | | | | | Treatment | | | | |
| | Height to flag (mm) | | | | | | | | |
| | | | | | | 398.3 ± 9.8 | 292.5 ± 5.9 | 220.7 ± 4.0 | |
| | | | | | | | p < 0.001 | p < 0.001 | p < 0.001 |
| | | | | | | | \*\*\* | \*\*\* | |
| | | | | | | a | b | c | |
| | | | | | | | | | |
| | Length auricle to ligule (mm) | | | | | | | | |
| | | | | | | 167.8 ± 4.7 | 132.1 ± 3.7 | 96.6 ± 3.7 | |
| | | | | | | | p < 0.001 | p < 0.001 | p < 0.001 |
| | | | | | | | \*\*\* | \*\*\* | |
| | | | | | | a | b | c | |
| n=24 | | | | | | | | | |

## Slide 9
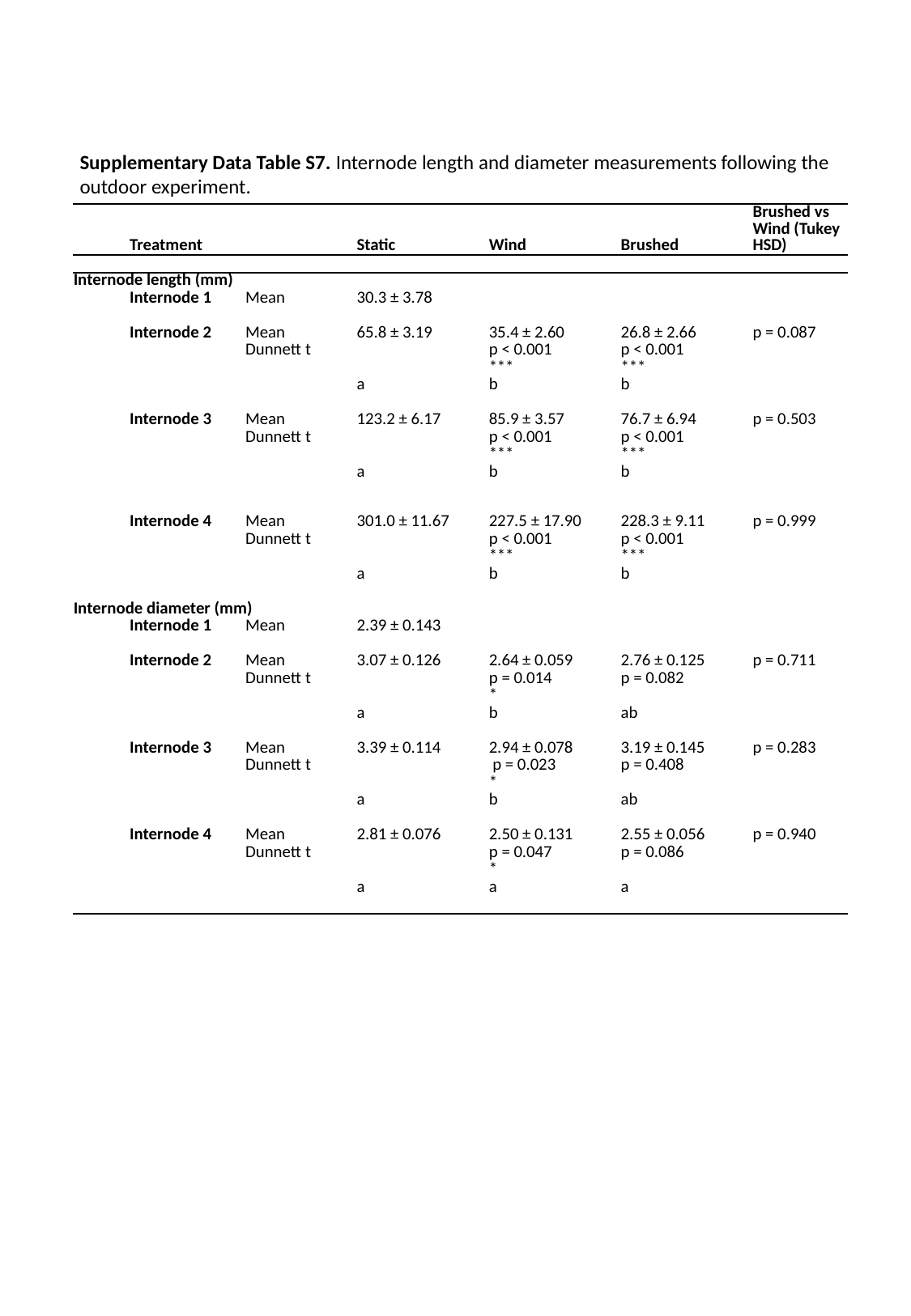

Supplementary Data Table S7. Internode length and diameter measurements following the outdoor experiment.
| | Treatment | | Static | Wind | Brushed | Brushed vs Wind (Tukey HSD) |
| --- | --- | --- | --- | --- | --- | --- |
| | | | | | | |
| Internode length (mm) | | | | | | |
| | Internode 1 | Mean | 30.3 ± 3.78 | | | |
| | | | | | | |
| | Internode 2 | Mean | 65.8 ± 3.19 | 35.4 ± 2.60 | 26.8 ± 2.66 | p = 0.087 |
| | | Dunnett t | | p < 0.001 | p < 0.001 | |
| | | | | \*\*\* | \*\*\* | |
| | | | a | b | b | |
| | | | | | | |
| | Internode 3 | Mean | 123.2 ± 6.17 | 85.9 ± 3.57 | 76.7 ± 6.94 | p = 0.503 |
| | | Dunnett t | | p < 0.001 | p < 0.001 | |
| | | | | \*\*\* | \*\*\* | |
| | | | a | b | b | |
| | | | | | | |
| | Internode 4 | Mean | 301.0 ± 11.67 | 227.5 ± 17.90 | 228.3 ± 9.11 | p = 0.999 |
| | | Dunnett t | | p < 0.001 | p < 0.001 | |
| | | | | \*\*\* | \*\*\* | |
| | | | a | b | b | |
| | | | | | | |
| Internode diameter (mm) | | | | | | |
| | Internode 1 | Mean | 2.39 ± 0.143 | | | |
| | | | | | | |
| | Internode 2 | Mean | 3.07 ± 0.126 | 2.64 ± 0.059 | 2.76 ± 0.125 | p = 0.711 |
| | | Dunnett t | | p = 0.014 | p = 0.082 | |
| | | | | \* | | |
| | | | a | b | ab | |
| | | | | | | |
| | Internode 3 | Mean | 3.39 ± 0.114 | 2.94 ± 0.078 | 3.19 ± 0.145 | p = 0.283 |
| | | Dunnett t | | p = 0.023 | p = 0.408 | |
| | | | | \* | | |
| | | | a | b | ab | |
| | | | | | | |
| | Internode 4 | Mean | 2.81 ± 0.076 | 2.50 ± 0.131 | 2.55 ± 0.056 | p = 0.940 |
| | | Dunnett t | | p = 0.047 | p = 0.086 | |
| | | | | \* | | |
| | | | a | a | a | |
| | | | | | | |

## Slide 10
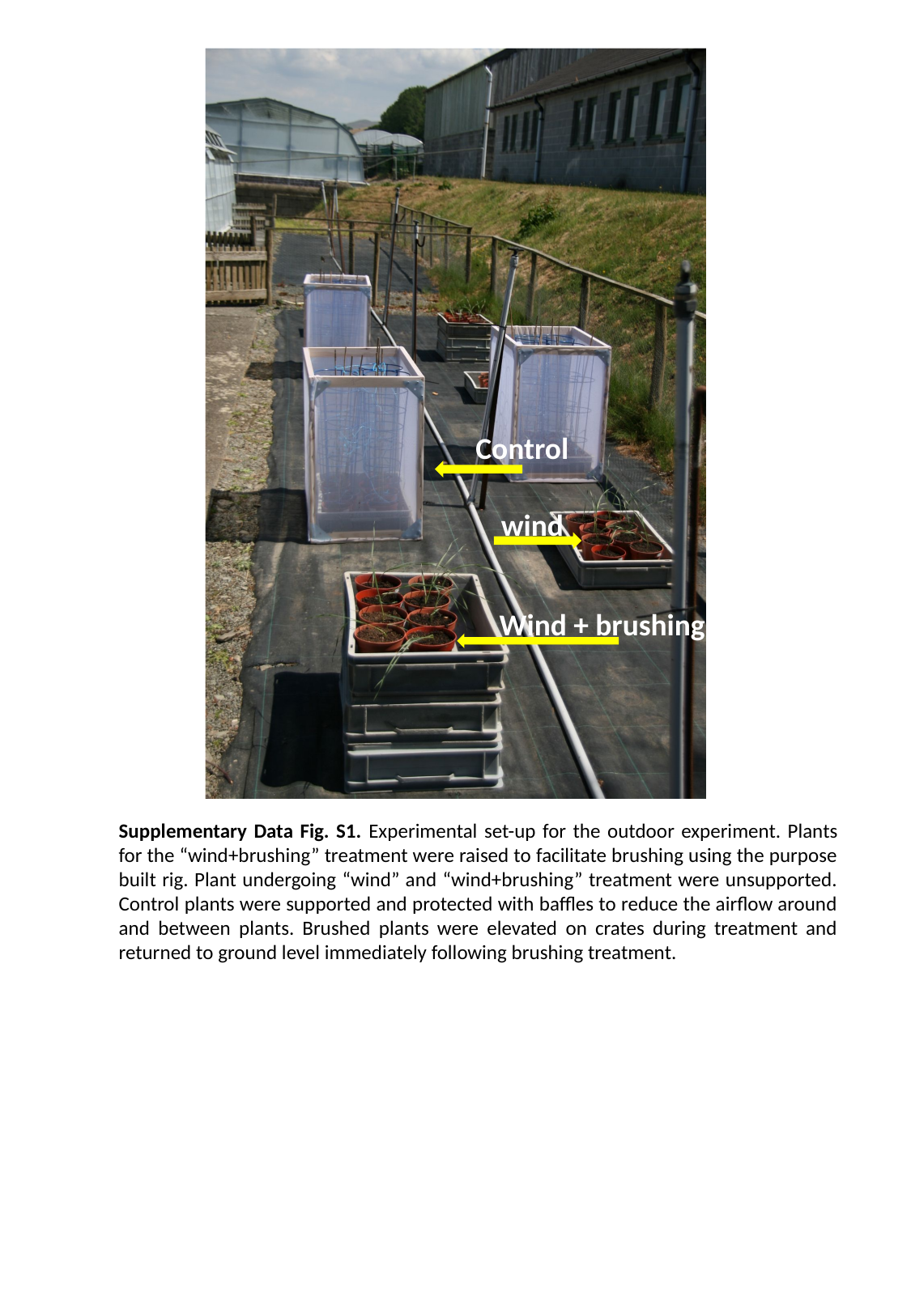

Control
wind
Wind + brushing
Supplementary Data Fig. S1. Experimental set-up for the outdoor experiment. Plants for the “wind+brushing” treatment were raised to facilitate brushing using the purpose built rig. Plant undergoing “wind” and “wind+brushing” treatment were unsupported. Control plants were supported and protected with baffles to reduce the airflow around and between plants. Brushed plants were elevated on crates during treatment and returned to ground level immediately following brushing treatment.

## Slide 11
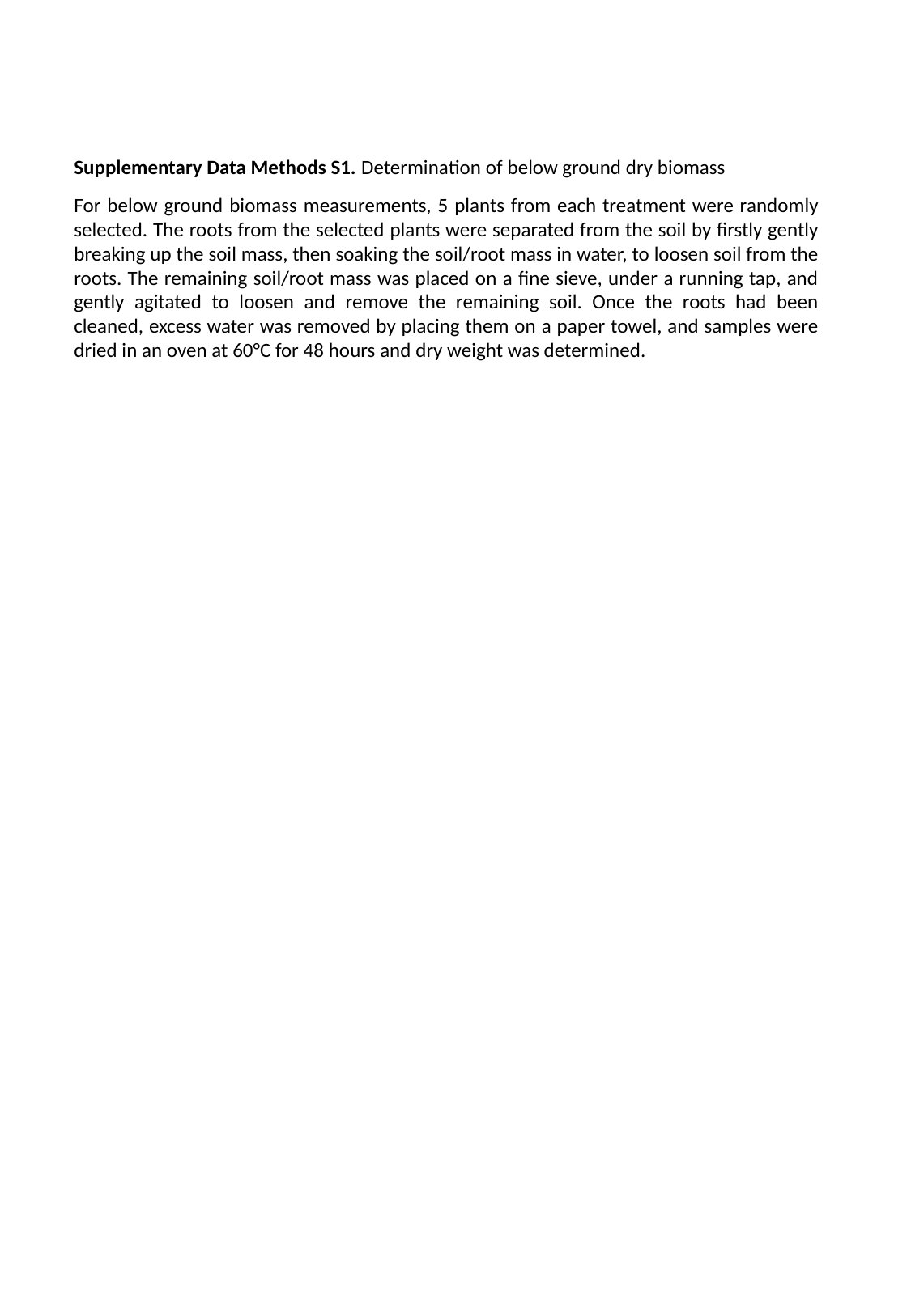

Supplementary Data Methods S1. Determination of below ground dry biomass
For below ground biomass measurements, 5 plants from each treatment were randomly selected. The roots from the selected plants were separated from the soil by firstly gently breaking up the soil mass, then soaking the soil/root mass in water, to loosen soil from the roots. The remaining soil/root mass was placed on a fine sieve, under a running tap, and gently agitated to loosen and remove the remaining soil. Once the roots had been cleaned, excess water was removed by placing them on a paper towel, and samples were dried in an oven at 60°C for 48 hours and dry weight was determined.

## Slide 12
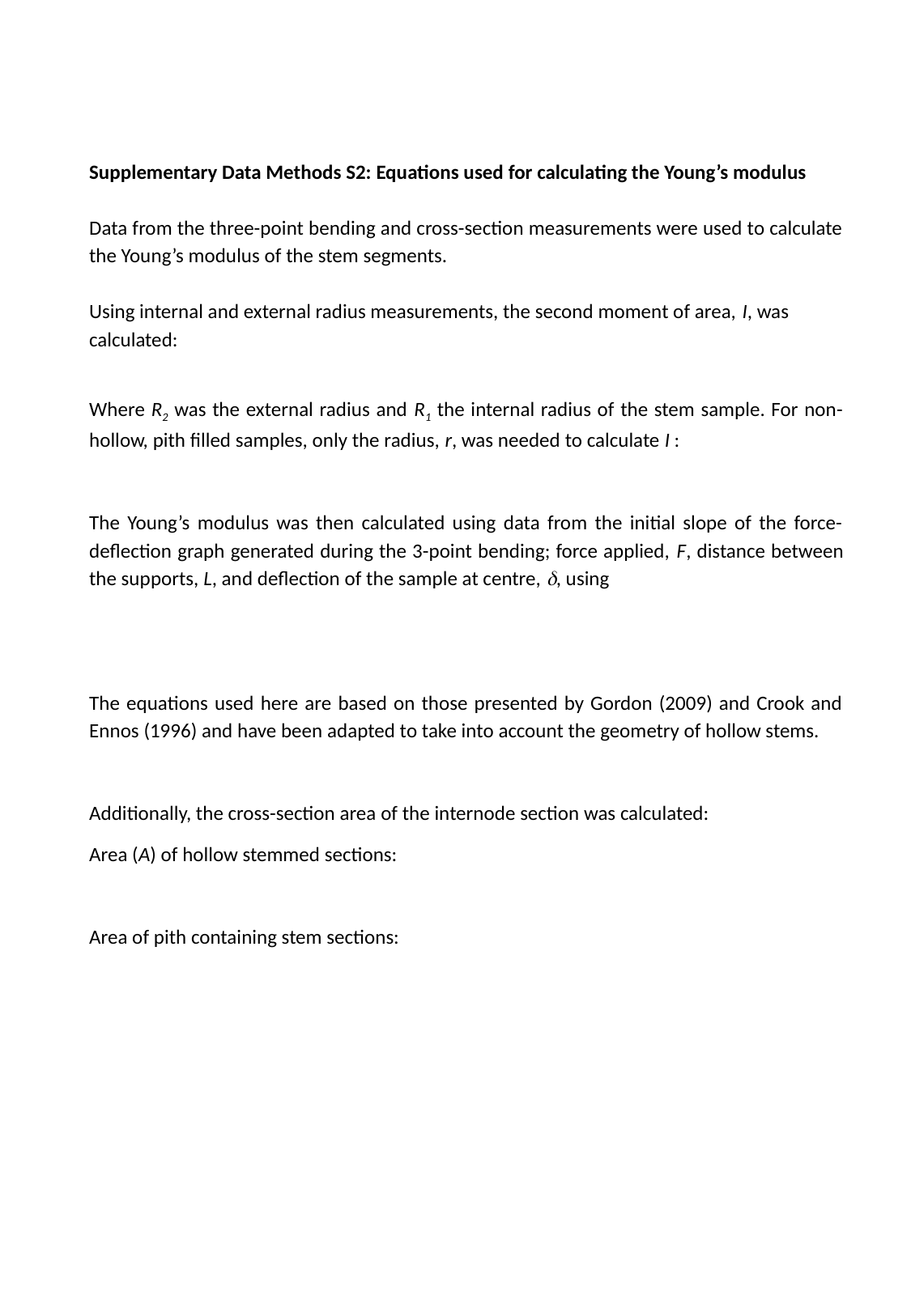

Supplement: mcab070_suppl_Supplementary_Material [file mcab070_suppl_supplementary_material.pptx]
